# Psychosocial–Spiritual Experiences and Outcomes in Parents of Children with Type 1 Diabetes Mellitus from the Middle East and North Africa Region: A Systematic Review

**DOI:** 10.1155/2024/6111661

**Published:** 2024-07-12

**Authors:** Mariam Asaad, Haya Abu Ghazaleh, Vasiliki Tzouvara, Xiaoyan Zhao, Jackie Sturt

**Affiliations:** King's College London, London, UK

## Abstract

**Background:**

Type 1 diabetes mellitus (T1DM) is prevalent in the Middle East and North Africa (MENA). Parents of children or young people (CYP) with T1D experience shock, devastation, guilt, and societal blame, which impact both physical and psychosocial–spiritual aspects of their lives. However, our knowledge of the breadth of these psychosocial–spiritual experiences and how they are assessed is limited.

**Aim:**

(1) To examine the diabetes-specific psychosocial experiences of parents of CYP with T1D in the MENA region; (2) to assess the person-reported outcome measures (PROMs) that measure the psychosocial–spiritual outcomes in this population; and (3) to assess their reliability and validity.

**Materials and Methods:**

A systematic review methodology was implemented using the preferred reporting items for systematic reviews and meta-analyses (PRISMA) guidelines. Ovid MEDLINE, Embase, APA PsycINFO, CINAHL, and Global Health databases were searched for relevant articles. A narrative synthesis approach was used for data analysis.

**Results:**

Twenty-three studies were included. We identified four categories: (1) spiritual functioning, parents' ability to accept and cope with their CYP's condition, (2) psychological functioning, parents' emotional distress due to insufficient diabetes-related knowledge and skills, (3) social functioning, describing financial challenges, social support experiences, and cultural concerns faced by parents, and (4) physical functioning, parents' struggle with sleep deprivation. Our results revealed methodological and conceptual limitations of the current tools measuring these experiences. Some of the limitations of this review are (1) heterogeneity in the tools captured perhaps some but not all domains of the parents' psychosocial experiences, (2) only English studies were included, as no Arabic studies were found.

**Conclusion:**

Our studied population experiences psychosocial–spiritual distress by managing the condition of their CYP and needs culturally specific psychosocial–spiritual support. Further studies are needed to develop a new measure to specifically assess the psychosocial–spiritual outcomes of this population.

## 1. Introduction

The prevalence of type 1 diabetes mellitus (T1DM) among children or young people (CYP) is increasing in the Middle East and North Africa (MENA) region [1]. According to the IDF Diabetes Atlas [1], the prevalence rate of T1DM in children and adolescents in the MENA region is estimated around 200,000 cases/year compared to 300,000 cases/year in Europe. The incidence rate has increased sharply in the MENA region from 13,000 cases/year in 2019 to 26,000 cases/year in 2021 while in Europe the incidence rate has risen from 25,000 cases/year in 2019 to 32,000 cases/year in 2021 [1, 2]. Research from both the MENA region and Europe suggests that the diagnosis of a child or young person (CYP) with T1DM is a life-changing experience for the family and requires adaptation to a new life situation [3]. Feelings of shock, devastation, guilt, and blame, and a learning curve that parents of CYP undergo with a diagnosis of T1DM have a significant impact on both physical and psychosocial aspects of their life [3, 4, 5]. The mental health, well-being, and adaptation of a parent to the diagnosis of their child with T1DM is known to impact the mental health and diabetes outcomes in their children living with the condition [6, 7, 8, 9]. While research which has looked at the psychological impact of T1DM has increased globally over the past few years [10, 11, 12], a focus on the spiritual impact has not been considered. Spirituality is defined by the National Cancer Institute as a deep, often religious, feelings, and beliefs, including a person's sense of peace, purpose, connection to others, and beliefs about the meaning of life [13]. Spirituality can help people bypass their realities by relying on a higher power to get through difficult and challenging times [14]. A qualitative study by Asaad et al. [3] shed light on this by underscoring the important role spirituality played in mothers' ability to emotionally cope and accept their CYP's diabetes, therefore highlighting that the psychosocial–spiritual consequences of living with T1DM of parents is under researched in the MENA region.

The American Diabetes Association (ADA) defines the psychosocial aspects of diabetes as complex, environmental, social, behavioral, and emotional factors that influence living with diabetes [15]. Thus, it is generally acknowledged that comprehensive healthcare should not only consider a person's biological needs but also their psychological and social [3, 16, 17]. In this way, effective, individualized treatment plans which consider the contextual factors that impact a person's health can be developed [18]. Yet, “psychosocial” aspects of diabetes care are often overlooked and not integrated into standard diabetes care [15, 16, 19].

Previous systematic reviews which have looked at the experiences of parents of CYP with T1DM suggest the need for annual screenings of parents of CYP with T1DM to assess levels of psychological distress, in order to address their mental health needs and provide appropriate support [20, 21, 22]. While the findings of these reviews may be applicable to the MENA region, none have focused on any specific cultural needs of CYP and their parents which have been identified as having a powerful influence on how they view and manage their health conditions [19, 23].

Putting the person or patient at the center of clinical care has led to the development of person-/patient-reported outcome measures (PROMs), which are key to evaluating the needs of patients and impact of care interventions. As the psychological and emotional burden of living with diabetes is often under-reported, the use of diabetes-specific PROMs can help assess any psychosocial–spiritual problems and allow the healthcare team to address them [24]. However, many of the existing diabetes-specific validated tools were developed for use in a Western context and while they focus on quality of life; they lack important constructs such as culture or spiritual/religious beliefs which can be important to certain populations in the MENA region [25]. To date, there is no research that has looked at the psychosocial–spiritual experiences of parents with CYP with T1DM in the MENA region, or the most optimal way to capture such outcomes in this population.

## 2. Aim and Research Questions

Therefore, the aim of this systematic review sought to answer the following questions:

- What are the psychosocial experiences and outcomes of parents caring for CYP with T1DM from the MENA region?
- What are the PROMs that have been used to measure psychosocial outcomes in this population?
- What is the reliability, validity, and cultural relevance of these PROMs for people from the MENA region?

## 3. Materials and Methods

A protocol for the systematic review was developed and registered with PROSPERO (register number: CRD42022355171). The review methods and presentation of results followed the 2020 Preferred Reporting Items for Systematic Reviews and Meta-Analyses (PRISMA) guidelines [26]. In addition, the COSMIN COnsensus-based Standards for the selection of health Measurement INstruments (COSMIN) checklist [27] was used to evaluate reliability, validity, and cultural relevance of the included PROMs.

### 3.1. Search Strategy and Eligibility Criteria

The PEO (population, exposure, outcome) framework [28] was used to develop the research questions and the search strategy for this review, including MeSH (Medical Subject Headings) terms and relevant search terms. Various terms relating to “parents and family” (population) “Type 1 diabetes” (exposure) and “psychological stress/distress and emotional well-being” (outcomes) were combined using Boolean operators as appropriate. The full search strategies for each database are available in Table S1. The following databases were searched on December 15, 2023, with automated alerts/notifications of new articles: Ovid MEDLINE (1946 to December 15, 2023), Embase, APA PsycINFO, CINAHL, and Global Health. The Cochrane database and PROSPERO register were searched for similar recent systematic reviews on the topic, but none were found. In addition, reference lists of eligible studies were searched, and gray literature including conference proceedings and theses were searched and papers from these were identified. No date limit was applied, but only studies conducted in English and/or Arabic were included.

### 3.2. Inclusion and Exclusion Criteria

Explicit selection criteria were established before the screening process, adapted from the PEO framework [28].

Inclusion criteria were as follows:

- Participants–parent/caregiver of CYP (1–18 years) with T1DM from MENA region.
- Exposure having a CYP with T1DM.
- Studies with data on psychosocial experiences and outcomes of parents of CYP with T1DM outcomes (including but not limited to well-being, quality of life, anxiety, distress, stress). Experience data could be collected qualitatively or through quantitative PROMs.
- All forms of study design (quantitative, qualitative, and mixed methods).

  Exclusion criteria: Participants whose children had an additional coexisting long-term physical condition (e.g., cystic fibrosis, mental ill health) as it would be challenging to assess the contribution of T1DM alone to their parental experience. Studies which only reported child's experience but not the parent's perspective.

### 3.3. Screening

Screening involved three stages: (1) identified papers were screened by abstract and title, (2) full-text article screening was conducted, and (3) the reference lists of the included studies were also screened for eligibility. Screening of titles and abstracts was completed by the first author (MA) with 20% of the initial screening of titles and abstracts reviewed by a second member of the research team (HA) adding to the reliability and validity of the process [29]. Discrepancies were resolved by a third reviewer (VT).

### 3.4. Data Extraction

Data extraction was supported by using Covidence [30] and data were exported into an Excel file. The data extraction template was pilot tested on two papers and its domains were found to be appropriate. Data extracted were authors, country, aims/objectives, sample size, gender, age, setting, type and duration of diabetes, study design, sampling method, data analysis, psychosocial experiences and experience outcome measurements, psychosocial constructs of outcome measurements, and methods for cultural adaptation. In papers where data related to children were reported, only data related to parents were extracted.

### 3.5. Quality Assessment of Individual Studies

The included articles were critically appraised by two members (MA, VT) of the research team using the appropriate tools. Quantitative studies were appraised using the appraisal tool for cross-sectional studies (AXIS) [31], the Joanna Briggs Institute (JBI) appraisal tool for quasi-experimental study designs [32], and the JBI appraisal checklist for case-control studies [33]. Qualitative studies were appraised using the JBI tool for qualitative studies [34]. The detailed findings from the critical assessment are included in Tables S5, S6, S7, and S8.

#### 3.5.1. Strength of Evidence

The strength of the evidence in the findings of the included studies was assessed by two members of the research team (MA, HA), and discrepancies were resolved by a third member of the team (XZ). Grading of recommendations assessment, development, and evaluation (GRADE) was used to assess the quality of the evidence derived from quantitative studies [35, 36]. In addition, the GRADE-CERQual (Confidence in the Evidence from Reviews of Qualitative research) approach was used to assess how much confidence could be placed in findings from qualitative studies [37].

#### 3.5.2. Method for Assessment of Content Validity of PROMs

The COSMIN checklist an established checklist for assessing measurement properties checklist [38] was used to assess the PROMs against their content validity [27].

### 3.6. Data Synthesis

Due to the heterogeneity in the study designs of the included studies, a meta-analysis was not feasible; thus, a narrative approach was used to synthesize and pool data and evidence into categories and patterns [39, 40, 41]. The analysis involved three steps: (1) develop a preliminary synthesis, by organizing findings to describe patterns; (3) explore relationships in data to consider factors that explain differences across studies; and (4) assess robustness of synthesis by drawing conclusions [41].

## 4. Results

**Figure 1:**
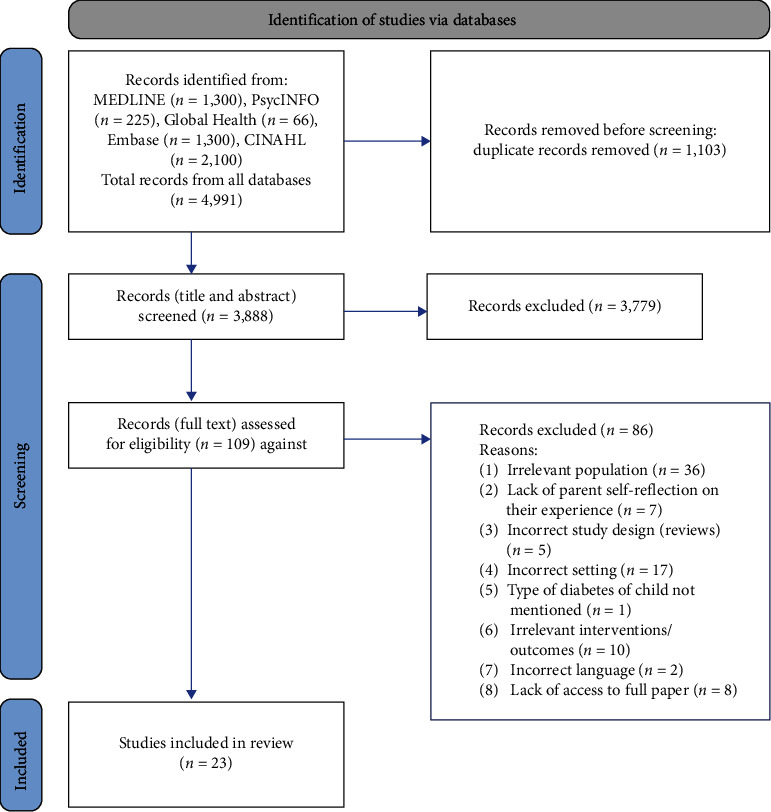
PRISMA diagram of identified studies through database searches.

The search identified 4,991 citations. After the removal of duplicates, 3,888 remained. After initial screening of titles and abstracts, 109 studies were identified as potentially relevant. Full-text screening identified 23 studies that met the inclusion criteria (Figure 1).

### 4.1. Overview of Study Characteristics

**Figure 2:**
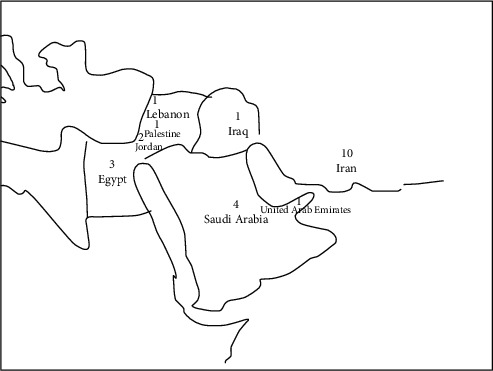
Countries of study and number of studies conducted in each country.

The studies were conducted across eight countries from the MENA region (Figure 2), Iran [42, 43, 44, 45, 46, 47, 48, 49, 50, 51], Saudi Arabia [3, 52, 53, 54], Egypt [55, 56, 57], Jordan [58, 59], Iraq [59], Lebanon [60], Palestine [4], and United Arab Emirates [61]. Nine studies were qualitative, and 14 used quantitative methodologies: cross-sectional (*n* = 11), case-control (*n* = 2), and quasi-experimental (*n* = 1) study designs. The total number of participants in the qualitative studies was 112, and the total number in quantitative studies was 1,610. Mothers made up 80%–100% of the individuals recruited, and participants' age ranged from 25 to 58 years of age.

Data collection methods were predominantly questionnaires (*n* = 14) and interviews (*n* = 9).

After the extraction of the data, a summary of the included studies was placed in Table 1.

### 4.2. Quality of Included Studies

The quality of the included studies varied significantly by design. Three cross-sectional studies did not share their methods of statistical analysis [47, 51, 62]. Methods of sampling were unclear in seven cross-sectional studies [43, 47, 51, 52, 59, 60, 62], and none provided data about nonresponders. In three cross-sectional studies, it was difficult to ascertain if they had checked the internal consistency or reliability of the PROMS used in their study [52, 60, 62]. One qualitative study had a very small sample size (*n* = 4) [61]. In two case-control studies, it was difficult to ascertain if the exposure period was long enough to be meaningful [44, 54]. For the quasi-experimental study, the comparison group was unclear, and it was difficult to ascertain if follow-up period was complete as this was not explicitly mentioned [50]. For the GRADE assessment, all of the included quantitative studies were observational studies, and none were randomized controlled trials (RCTs) therefore they all started with a “low” rating and were either upgraded or downgraded or remained “low.” Imprecision was present in most outcomes due to the small sample sizes of the included studies. As for the GRADE-CERqual assessment, many of the outcomes also were only found in one study and some had thin data, meaning not enough quotations, to support the outcome therefore impacting the adequacy of the data. Further findings from this assessment and the critical appraisal of the individual studies are included in Tables S5, S6, S7, S8, S9, and S10.

## 5. Narrative Findings

**Figure 3:**
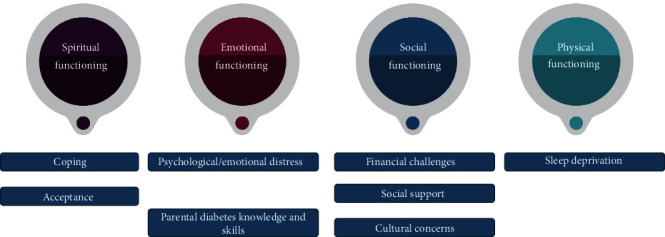
Categories and subcategories of studies.

Four main categories and several subcategories that described the nature of the impact that the child's diabetes had on parents were identified, as shown in Figure 3.

## 6. What Were the Psychosocial Experiences of Parents with CYP with T1DM from the MENA Region?

### 6.1. Category 1: Spiritual Functioning

Faith permeated eight of the qualitative studies as a method of coping for the parents and a method of acceptance of their CYP's diagnosis.

#### 6.1.1. Coping with Diagnosis

Parents attributed the diagnosis of their child to “God's will” and that it was their “child's destiny” thus easing the initial period after the shock of the diagnosis [4, 57, 58, 61]. In three qualitative studies, prayer gave parents a sense of hope [3, 48, 61]. Parents in four studies expressed their coping by uncovering the silver lining of their CYP's diagnosis. In two qualitative studies, parents adopted an optimistic approach to being happy making the atmosphere at home happier [48, 49]. In another two qualitative studies mothers found that the diagnosis of their CYP strengthened their relationship with their spouse, family, and friends. Whereby their faith was tested at diagnosis and through the healing of time and their practice of prayers and prayers from the people surrounding them, they were able to cope [3, 61].

#### 6.1.2. Accepting the Condition

In nine studies parents shared their experience of acceptance of their CYP's condition. Mothers in three qualitative studies shared how they eventually adjusted, accepted, and normalized their CYP's condition [3, 46, 61]. In one qualitative study, the parent's faith helped them accept their child's diagnosis [57], and in another qualitative study, a mother felt it was a divine exam thus helping her accept the diagnosis [46]. Acceptance, meaning spiritual submission to unfortunate events, was the method of accepting their CYP's diagnosis for mothers in a cross-sectional study conducted in Egypt [55]. The concept of destiny was evident in two qualitative studies which helped parents accept the diagnosis [58, 61]. A quote from a mother in a qualitative study “for me it was a lot of prayer and being close to God, eliminated a need for psychological support” summarized the role of faith in her life whereby it eliminated her need for psychological support [3].

As part of the parents' attempt to accept their CYP's condition, they tried to understand why their CYP got diabetes. In one qualitative study, a mother attributed the cause of her daughter's condition to the evil-eye because she used to post pictures of her daughters on social media without saying the special prayers to ward off envy [3]. Time and the spiritual connection with God through prayer played an essential role in helping mothers in two qualitative studies reach a stage of acceptance and normalization [3, 61].

### 6.2. Category 2: Psychological Functioning and Education

The parents experienced psychological and emotional distress when their CYP was diagnosed with T1DM. However, when they overcame the shock of the diagnosis, they were able to empower themselves with diabetes-related knowledge and skills.

#### 6.2.1. Emotional Distress

Most qualitative studies after the period of diagnosis documented that parents experienced feelings of shock, emotional devastation, and difficulty facing the news [3, 45, 46, 49, 51, 57, 58, 61]. A case-control study conducted in the Kingdom of Saudi Arabia (KSA) found that the mothers of CYP with T1DM experienced significantly higher levels of nervousness, irritability, poor concentration, and fear than mothers of healthy CYP indicating higher stress in mothers of CYP with T1DM [54]. Whilst multiple studies have indicated that parents of CYP with T1DM experience depression, anxiety and stress; however, these symptoms were shown to improve after a year of diagnosis in one study [51]. Emotional distress was assessed in two cross-sectional studies conducted in KSA. One study by Aldubayee et al. [53] assessing “emotional distress” in parents found that high levels of emotional distress were associated with the long-term impact of the condition and the uncertainty related to future complications. The “emotional functioning” subscale of the Health-Related Quality of Life (HRQoL) scale was used; and the authors found that worry was related to emotional distress [52].

In four cross-sectional studies parents reported depressive symptoms, anxiety, and stress when dealing with the difficulties of day-to-day management of their CYP's condition [47, 53, 59, 62]. Diabetes management activities were found to increase parents' stress levels as noted in two cross-sectional studies [42, 53]. Specifically fear of hypoglycaemia was assessed in one cross-sectional study, where they found that mothers were more prone to experiencing emotional distress in relation to managing their CYP's condition and preventing hypoglycaemia [42]. High levels of “emotional distress” were also reported by mothers and fathers in a cross-sectional study from Iran in relation to managing their child's condition [42].

#### 6.2.2. Parental Diabetes Knowledge and Skills

In seven studies, parents empowered themselves with knowledge and skills to help manage their CYP's condition. In four qualitative studies, mothers tried to seek information through social media support platforms or local diabetes care centers [3, 45]. In several cross-sectional studies, knowledge was associated with better ability to manage their CYP's condition, and an enhanced state of self-efficacy and the ability to cope with stress [42, 43, 55, 56]. In addition, the more information parents had the better their coping outcomes [43].

### 6.3. Category 3: Social Functioning

#### 6.3.1. Financial Consequences of Diabetes

One of the biggest challenges for parents with a CYP with T1DM was the financial impact of the condition on families from studies conducted in low- to medium-income countries (LMIC) such as Iran, Egypt, Lebanon, and Palestine [4, 45, 49, 56]. In five qualitative studies, parents discussed the financial impact and burden of T1DM [4, 45, 49, 57] including having inadequate funds to buy glucose monitoring strips to support her child's glycaemic control [49], or secure professional psychological support for her child [45]. Indeed, insufficient income was also found to have a statistically significant impact on parents' mental health and impair their ability to perform social activities in two studies [4, 47], and the financial burden of the disease was found to have a significant impact on mothers of CYP with T1DM in Iran [44]. In Egypt, a higher household income was associated with greater access to resources and better diabetes-related health behaviors [56]. A cross-sectional study from Lebanon also found that 59% of parents allocated had to specifically allocate a budget for their CYP's diabetes treatment [60].

#### 6.3.2. Experiences of Social Support

In several studies, mothers spoke about the impact of their CYP's condition on their social lives and how they felt guilty leaving their child [4, 46, 48] and some experienced separation anxiety [45]. A cross-sectional study from Iran that used the family quality of life (FQoL) measure found that mothers had to decline social occasions to care for their CYP [44]. In a qualitative study from Jordan, the high prevalence of diabetes in the community helped a mother accept her CYP's condition as “the disease of the century” hence helping her cope [58].

Parents had varied experiences with support from family, friends, and healthcare professionals (HCPs) [3, 45, 46, 48, 61]. Many of these experiences stemmed from their cultural context where illness should be hidden to avoid societal stigma and blame. In one qualitative study, parents sought positive social connections and support and disconnected from negative unsupportive people [48]. In four qualitative studies, parents reported positive support from family [3, 45, 48, 61], HCPs [48, 57, 61], and schools [61]. However, parents described the lack of support from HCPs [3, 45]. A mother from one of these studies described how the HCPs had undermined her concern of a genetic link in terms of her other children getting T1DM after the diagnosis of her second child with the condition [3]. Parents in three qualitative studies reported a distinct lack of support from schools [3, 4, 61] with examples of discrimination such as not being allowed on school trips [4].

#### 6.3.3. Cultural Concerns around T1DM

The stigma of illness was a common pattern arising from parents' experience of having a CYP with T1DM. In two qualitative studies, mothers kept the news of the diagnosis a secret, because they feared facing blame from their families and social circles [4, 61]. Mothers faced blame from the wider family for being responsible for their CYP's illness [46]. In another qualitative study, a mother expressed feeling upset by societal pity when people came to learn of her CYP's condition [4]. Similarly, a cross-sectional study that assessed the quality of life found that the domain of “status,” meaning respect and support from society was significantly (*p*=0.0003) lower in mothers of CYP with T1DM [44]. On the other hand, a mother from another qualitative study shared how people always prayed for her CYP [4].

Another dominant cultural concern of parents across many qualitative studies was about the impact of their CYP's condition on their ability to get married and have children, particularly in females [4, 46, 49, 57, 61]. In one qualitative study, the parents emphasized their daughter's need to focus on her studies due to her slim prospects for marriage [4].

### 6.4. Category 4: Physical Functioning

#### 6.4.1. Sleep Deprivation

One study showed an increase in reported insomnia among mothers of CYP with T1DM as a result of monitoring their CYP's blood glucose levels at night when they were more prone to hypoglycaemia. This was assessed using the Rahim Anxiety Depression (RAD) scale, and they found significantly increased insomnia, fatigue, poor concentration [54]. Similarly, a phenomenological study conducted in KSA showed that mothers experienced insomnia whereby a mother described how none of the HCPs warned her about the sleep difficulties that lay ahead [3].

## 7. What PROMs Have Been Used to Measure These Psychosocial Experiences in This Population?

Thirteen different PROMs were identified from the studies, and the most widely used were *n* = 2 the Parenting Stress Index (PSI) and the Health-Related Quality of Life (HRQoL). A list of the PROMs and their uses are listed in Tables 2 and S2.

A full description of the outcomes and constructs that were assessed in this review is shown in Table S3.

## 8. What Is the Reliability and Validity and Cultural Relevance of These PROMs for People from the MENA Region?

The COSMIN checklist highlights the psychometric properties of the PROMs included in this review (Tables 3 and S4).

## 9. Discussion

This review has identified that parents in the MENA region experience significant distress, stress, anxiety, worry, and potentially decreased quality of life in relation to their CYP's condition. In addition, it has highlighted the importance of cultural and spiritual constructs for this population which not only influence the way they experience diabetes-related psychosocial–spiritual distress but also the approaches they use to cope and adapt to their CYP's condition.

Parents reported emotional distress in relation to the day-to-day management of their CYP's condition [3, 42, 45, 46, 48, 54, 56, 57, 58, 59, 61, 62]. These reactions to childhood long-term conditions such as cerebral palsy, congenital heart disease, epilepsy, and cancer are relatively common across different conditions [66, 67, 68, 69] and also mirror other reviews of parental experiences of caring for a CYP with T1DM [20, 21, 22]. Financial burden of care was a particular source of distress for parents LMIC [4, 45, 49, 56]. This was also found in a global systematic review [21] which described the financial implications of raising a child with T1DM, due to providing diabetes-related supplies.

CYP's future with regard to diabetes-related complications and their ability to lead normal lives as adults was a major concern [4, 45, 46, 48, 52, 57, 60, 61]. This was a result of cultural beliefs and lack of knowledge in relation to diabetes, fears, and misconceptions, such as anxiety regarding their daughter's ability to marry and have children [4, 45, 48, 56, 57, 61].

Parents' unconscious beliefs impacted the way they accepted and coped with their CYP's condition. A study by Asaad et al. [3] found that mothers felt guilty for feeling angry or sad when their CYP was diagnosed with the condition. However, religious practices such as reciting the Quran or repeating prayers were found to be an effective method of coping with the distress of their CYP's diagnosis in several studies based in Muslim countries [3, 61, 70]. This mirrors findings from a review by Alkhateeb et al. [71] where religious coping was a method that resulted in strength and resilience in parents of children with autism spectrum disorder (ASD).

Lack of appropriate skills such as not knowing how to give an insulin injection or check a blood glucose level and feeling ill-prepared for managing their CYP's condition on their own after discharge from hospital was found [3, 45]. There was a clear need for diabetes-specific information, diabetes-related skills training, and emotional support for parents. Indeed, parents' greater need for emotional support and clear training to care for their children has been reported in studies from the MENA region including parents of children with other long-term conditions [72, 73]. Professionals can help with this process, by explaining the biological causes of T1DM, addressing any misplaced feelings of guilt or shame, and updating parents on new advancements in diabetes technology to improve glycaemic control and lower complications. HCPs need to adapt their approach to better reflect the cultures of CYP and their parents by showing empathy, humanity, kindness, and by making space for conversations on existential or spiritual questions during consultations.

The biopsychosocial–spiritual model stems from Engel's biopsychosocial model [16] which emphasized that healthcare must take into account the context in which the person lives, thus reconciling psychological and social factors into the biomedical model. Spirituality in Islam is inspired by both spiritual and physical entities that realize the person's needs [74]. The powerful conscious belief in prayer; a form of spirituality can give meaning to suffering, making it more bearable and manageable [3, 75].

It is important to consider alternative methods of support that encompass cultural constructs such as prayer and perhaps faith-related support groups. A previous review by Niazi and Kalra [76] looked at the diabetes communities in South Asia and emphasized the profound influence of Muslim community leaders in raising awareness about diabetes and methods of prevention. Such an approach could be encouraged through social media throughout the MENA region to prevent the spread of misinformation. Equally an increase in community awareness programs developed by HCPs in conjunction with community leaders could also help raise the awareness about the causes, symptoms of T1DM. Peer support or group interventions for parents and their CYP have been shown to be effective in improving knowledge, self-efficacy, and self-care [77, 78, 79]. Such approaches, where support is provided by people with shared local knowledge and cultural backgrounds, may be appropriate for people within the MENA region [14].

The lives and experiences of parents in the included studies of this review, though profoundly altered by diabetes, brought to light some of the positive outcomes of T1DM on them. Indeed, “diabetes” brought some families together whereby they supported each other through their belief and prayer [3, 61]. This is similar to a finding in an ongoing study [80, 81] where families came together to support each other and adopted a healthier lifestyle as a result of the diagnosis of their child with T1DM.

Only four of the PROMs from the included studies were diabetes-specific (HFS-P, SED-P, DKQ-24, and RSQ). Only one of the PROMs (RSQ) assessed diabetes-specific stress and SED-P assessed self-efficacy, but the other two (HFS-P, DKQ-24) did not assess the diabetes-specific psychosocial outcomes. Although some of the other widely used PROMs (PIP, PSI) did assess stress in parents of CYP with T1DM, they are not specifically designed for parents of CYP with diabetes [82, 83]. Even though a few (*n* = 3) of the PROMs in the studies demonstrated “adequate” validity and reliability, the lack of the assessment of the cross-cultural validity and the lack of inclusion of cognitive interviews bring into question the strength of the methodology of the included studies. Moreover, their lack of focus on the psychosocial–spiritual outcomes of diabetes adds doubt to their validity in the target population.

Finally, the findings of this review in part rely on the appropriateness of the PROMs used to capture parental experiences. The PROMs included in this review lacked the essential assessment of cross-cultural validity. Kūkea Shultz and Englert [84] argued that cultural validity should be the foundation for building validity and should be central to any traditional psychometric validity study to ensure sensitive and accurate measurement [84, 85]. The process of development of PROMs needs to be underpinned by robust guidelines such as those of COSMIN. The routine inclusion of an approach such as cognitive interviewing would be an ideal to complement psychometric approaches in PROM construction and will avoid building a weak foundation to the assessment of psychosocial–spiritual outcomes in this population. Failing to involve patients or family members in the design and development process of the content of PROMs is poor practice [86]; as their input is necessary to identify constructs that are culturally relevant. When developed in this way, PROMs will reliably identify areas of diabetes management which parents are struggling with and enable the provision of targeted support.

## 10. Strengths and Limitations

This is the first systematic review to provide insights to assist with understanding the diabetes-specific psychosocial–spiritual experiences of parents of CYP with T1DM from the MENA region. It followed a transparent, evidence-based approach to study selection and data synthesis, such as the use of GRADE-CERQual. “Spirituality” was a finding of this review and was not used as a “search term” in the database searches, which perhaps could have led to the identification of further studies or not. A limitation of this review is that the heterogeneity in the tools captured perhaps some but not all domains of the parents' psychosocial experiences. In addition, the primary studies from which the review findings were derived were often poorly reported with a failure to include details on their analysis methods. When considering measurement tools and approaches to care which are appropriate for parents in the MENA region as a whole, broad recommendations may not reflect the many different cultural and economic factors which exist between countries in this region. A final caveat is that only studies in English were included although studies in Arabic were eligible, none were identified.

## 11. Conclusion and Implications for Practice

Parents of CYP with T1DM in the MENA region report significant physical, emotional, financial, social, and spiritual distress. As the majority of PROMs used in diabetes research to date have been developed for Western populations, they are unlikely to effectively capture the breadth of their experiences specifically anxiety, stress, and distress. Future research needs to focus on developing person-centered assessments which consider the cultural context of the region and are underpinned by a biopsychosocial–spiritual approach.

## Figures and Tables

**Table 1:** Summary of included studies.

Number	First author/year/country	Study design	Sample size/participant characteristics		Purpose	Method of data collection	Key findings
Quantitative studies							
1	Felimban et al. [54] 1998 (Saudi Arabia)	Case-control study	Study population: *n* = 110 mothers Age of mothers: Average age 33.46 ± 7.8 years Employment of parents: Not reported (NR) Education of mothers: Average years education 7.46 ± 5.54 years Age of fathers: NR	Education of fathers: Average school education was 9.73 + 6.70 years Marital status: NR Family income: NR Setting: Several hospitals and primary health clinics in Riyadh	To identify psychosocial effects on the mothers of children with T1DM	The Rahim Anxiety Depression (RAD) questionnaire Diabetes-specific PROM: no	The psychological symptoms of all the domains of the scale nervousness, irritability, insomnia, fatigue, fear, poor concentration, and indecision were found to be higher in mothers of CYP with T1DM (*p*=0.0002, *p*=0.0005, *p*=0.0002, *p*=0.0021, *p*=0.0002, *p*=0.0260, *p*=0.0391), respectively An increase in monitoring of blood glucose levels increased mothers' psychological distress

2	Talakoub and Nasiri [51] 2012 (Iran)	Quantitative observational study	Study population: *n* = 35 parents Age of parents: NR Employment of parents: NR Education of parents: NR Marital status: NR Family income: NR Setting The diabetes center of Amin Hospital of Isfahan University of Medical Sciences		To identify the affective reactions of parents during various stages of their children's disease	The Symptom Checklist-90 (SCL-90) questionnaire Diabetes-specific PROM: no	Depression reduced in parents from a mean of 17.43 1-month postdiagnosis to a mean of 3.6a year postdiagnosis (*p* < 0.01). Anxiety reduced from a mean of 11.2 1-month postdiagnosis to a mean of 2.05 a year postdiagnosis (*p* < 0.02)

3	Al Buhairan et al. [52] 2016 (Saudi Arabia)	Cross-sectional study	Study population: *n* = 315 mothers Age of mothers: NR Employment of mothers: 78.27% unemployed Education of mothers: 40% primary/no education 34% secondary education 25% university education Marital status: NR Family income: NR Setting: National Guard hospitals are national referral centers for chronic illnesses		To measure the impact T1DM on the family	(1) Peds Quality of Life Family Impact Module (Peds QL FIM) instrument (2) Health-related quality of Life (HRQoL) Diabetes-specific PROM: no	The adolescent's condition did not impact family functioning but did have a negative impact of parents' psychological well-being The greatest impact of HRQoL was “worry” about diabetes-related complications and treatment effectiveness

4	Saghaei et al. [50] 2017 (Iran)	Quasi-experimental design	Study population: *n* = 50 mothers *n* = 25 intervention group *n* = 25 control group Age of mothers: NR Employment of mothers: NR Education of mothers: All mothers had secondary to high school level education Marital status: NR Family income: NR Experimental group of mothers: Diabetes management training through cognitive behavioral therapy (CBT) sessions Control group of mothers: Did not undergo any training. Setting: Aram Consultation Center in Shahrekord City in Iran		To evaluate the effectiveness of a training course of cognitive behavioral stress management in glycaemic regulation in children with T1DM as well as in mental health of their mothers	(1) The Depression Anxiety Stress Scale (DASS) (2) The Parenting stress index (PSI). Diabetes-specific PROM: no The intervention included eight sessions related to educational diabetes management through CBT that lasted 2 hr. They were conducted over 2 months and included relaxation, anger/time management	The CBT stress management intervention reduced depression, anxiety, and stress (*p*=0.02) in mothers in the experimental group, but no changes were observed in the control group The effectiveness of CBT stress management intervention was significant (*p* < 0.05) in the experimental group
5	Amiri et al. [42] 2018 (Iran)	Cross-sectional study with psychometric study	Study population: *n* = 95 parents *n* = 60 mothers *n* = 45 fathers Mothers: Age of mothers: Age ranged from 25–49 years Employment of mothers: 75% unemployed Education of mothers: 65% of high school graduates Fathers: Age of fathers: Age ranged from 30–58 years Employment of fathers: 94.8% Education of fathers: 64% high school graduates employed Marital status: NR Family income: 88.5% monthly income under $800 Setting: The Gabric Diabetes Education Association (GDEA), Iran		To describe glycaemic control and investigate the fear of hypoglycaemia (FoH), emotional distress, and self-efficacy related to diabetes management in parents of CYP with T1DM	(1) Hypoglycaemia Fear Survey Parent version (HFS-P): worry related to hypoglycaemia (2) Pediatric Inventory for Parents (PIP): emotional distress (3) Self-Efficacy for Diabetes Scale Parent version (SED-P) Glycaemic control (HbA1c) 8.3% of children had optimal glycaemic control with and HbA1c level 10 years had less optimal glycaemic control Diabetes-specific PROM: yes (HFS-P), (SED-P)	The total score for fear of hypoglycaemia was higher (*p*=0.022) for mothers than fathers Parental stress was 51% above the midpoint in of mothers and 29.7%–40% above the midpoint in fathers. Indicating mothers experience more emotional distress in relation to their CYP's diabetes management Parents' sense of self-efficacy showed a cut-off level of 66, indicating an acceptable sense of self-efficacy

6	Mahfouz et al. [56] 2018 (Egypt)	Cross-sectional study	Study population: *n* = 92 mothers Mothers: 60.9% lived in urban areas 51.1% marriages were consanguineous Age of mothers: NR Employment of mothers: *n* = 78 unemployed *n* = 14 employed Education of mothers: *n* = 62 primary education *n* = 24 high school education *n* = 4 university education Employment of fathers: *n* = 25 unemployed *n* = 66 employed	Education of fathers: *n* = 56 primary school education *n* = 21 high school education *n* = 15 university degree Marital status: NR Family income: NR Setting: The diabetes clinic in the Minia Pediatric University Hospital, Egypt	To examine the relationship between mothers' knowledge related to diabetes management with perceptions of coping with diabetes-related distress	2 PROMs were used: (1) The Ways of Coping Questionnaire (WCQ) (2) The Diabetes Knowledge Questionnaire-24 (DKQ-24) HbA1c levels of the CYP were collected Diabetes-specific PROM: yes (DKQ-24) other PROM (WCQ) is not	The total knowledge scores for mothers of females were higher than mothers of males (*p* = 0.04). Positive correlation between the total knowledge scores and positive reappraisal, confrontation, and self-control (*r* = 0.28, *p*=0.001; *r* = 0.25, *p*=0.01; *r* = 0.24, *p*=0.02), respectively. The coping scale indicated mothers' coping was not related to glycaemic control. The increased mother's knowledge score with female CYP with T1DM could be related to their worries about their daughter's ability to marry due to the social stigma against their ability to conceive children. Father's education was linked to a higher income and yielded a higher total knowledge score (*p*=0.0001).
7	Noueri and Nassif [60] 2018 (Lebanon)	Cross-sectional study	Study population: *n* = 37 families Age of parents: NR Employment of mothers: 83.8% unemployed 16.2% employed Education of mothers: 40.5% primary education 43.3% high school 16.2% college education Education of fathers: NR Employment of fathers: NR Education of fathers: 35.2% primary education. 48.6% high school 16.2% college education	Marital status: NR Family income: 56.8% income of <$1,000 40.5% income of $100–$15,002.7% income of >$1,500 Setting: Not-for-profit institution for chronic childhood diseases	To assess the psychological effects and financial burden that parents have to deal with in their daily life	One questionnaire was used: It was self-developed to collect data related to the psychological and financial impact of T1DM on families and its oral complications on families. Diabetes-specific PROM: not clear	35.1% families felt there was no difference between raising a CYP with T1DM and healthy CYP 75.7% parents felt guilty about CYP's condition. 97.3% mothers felt the need to remain close to CYP with T1DM. 13.5% of parents aware of oral complications, 86.5% felt dental care was more important for CYP with T1DM 100% parents allocated a special budget for CYP's dietary requirements (*p*=0.012) 59.4% parents allocated a budget for treatment 10.8% parents struggled with treatment expenses 81% parents felt CYP's 's condition impacted work attendance

8	Aldubayee et al. [53] 2020 (Saudi Arabia)	Cross-sectional study	Study population: *n* = 390 parents Mothers: 95% of participants were mothers (*n* = 270) Age of parents: NR Employment of mothers: *n* = 58 employed *n* = 335 unemployed Education of mothers: *n* = 40 no education *n* = 107 primary *n* = 186 secondary *n* = 2 college Fathers: Employment of fathers: *n* = 343 employed *n* = 31 retired *n* = 3 unemployed	Education of fathers *n* = 12 no education *n* = 53 primary *n* = 171 secondary *n* = 156 college Marital status: *n* = 363 married *n* = 29 not married Family income: NR Living in Riyadh: *n* = 367 in Riyadh *n* = 25 not in Riyadh Setting: 5 diabetes clinics in government-funded hospitals	To evaluate the degree of parental involvement in routine care of their child with T1DM and to assess the levels of stress in parents caused by diabetes care	Pediatric Inventory for Parents (PIP) Diabetes-specific PROM: no	The highest level of stress was in the emotional distress domain with a mean of 26 (frequency) and 28.9 (difficulty). The highest mean in this domain was associated with the long-term impact of the disease and the uncertainty related to future complications Parents experienced less stress in medical care The diagnosis of their child with T1DM did not impact the families' ability to work. The level of stress was higher in separated parents but lower in parents who were employed There was a correlation between the age of the child and parental stress levels. The Spearman's correlation was −0.201 There was a correlation between glycaemic control (HbA1c levels) and parental stress levels. The Spearman's correlation was 0.379.

9	Shavaki et al. [43] 2020 (Iran)	Cross-sectional, mixed-methods psychometric study	Study population: *n* = 180 mothers Age of mothers: NR Employment of mothers: 80% unemployed Education of mothers: 43.3 % had an education Marital status: NR Family income: NR Setting: The Diabetes Association of Karaj and the Endocrinology and Metabolism Research Centre of Tehran		To investigate the factors associated with behavioral functions in mothers of children with T1DM based on the transactional stress and coping model	(1) The tool consisted of 50 questions and several constructs (2) The third part of the questionnaire used the Persian version of the Health Promoting Lifestyle Profile (HPLPII) Diabetes-specific PROM: no	Level of mother's education led to better health promotion behaviors (*p*=0.039). Management of stress and emotional responses had significant relationships with other structures (*p* < 0.05) Mothers who were more vulnerable to their CYP's condition had poor health-related behaviors The use of problem-focused coping methods was associated with better health-related behaviors
10	Hashemipour-Zavareh [44] et al. 2020 (Iran)	Cross-sectional study	Study population: *n* = 50 mothers in the intervention group (mothers of CYP with T1DM) *n* = 50 in the control group (mothers of healthy CYP) Age of mothers: Age from 20 to 60 years Employment of mothers: *n* = 19 employed *n* = 30 unemployed *n* = 11 students	Education of mothers: *n* = 10 elementary *n* = 14 high school *n* = 24 university Marital status: NR Family income: NR Setting: The endocrine and metabolism clinics in Isfahan City, Iran	To compare QoL for families with CYP with T1DM to those with healthy CYP from their mother's perception	Family Quality of Life quality scale was used (Persian version) Diabetes-specific PROM: no	Less respect from society was reported by mothers (*p*=0.0003) Social activities of mothers were impacted by CYP's condition (*p*=0.001) Ability to seek information was lower in mothers of CYP with T1DM (*p*=0.002) Mothers reported an inability to buy goods to prioritize care for CYP (*p*=0.013) Family finances and money were lower (*p*=0.010) in mothers of CYP with T1DM Love and affection were similar in both groups. Family life quality score was lower (*p*=0.002) in mothers of CYP with T1DM

11	Obaid et al. [62] 2020 (Iraq)	Cross-sectional study	Study population: *n* = 52 mothers Age of mothers: NR Employment of mothers: *n* = 48 unemployed *n* = 4 employed Education of mothers: *n* = 1 illiterate *n* = 19 primary education *n* = 13 secondary-college education	Marital status: *n* = 49 married *n* = 1 = divorced *n* = 2 widowed Family income: Average income of families $200–$600. Setting: NR	To assess burden of caring for children with T1DM upon mothers	Part 2 of the scale was self-developed and was made of 25 items related to the economical, psychological, and social burden of care Diabetes-specific PROM: not clear	(1) 86.5% of the mothers experienced a moderate level of burden of care (2) 61.5% of mothers experienced psychological burden of care (3) 51.9% of mothers experienced social burden of care (4) 40% of mothers expressed the high financial impact of the condition on their burden of care

12	Obeidat et al. [59] 2020 (Jordan)	Descriptive correlational design	Study population: *n* = 98 parents Mothers: *n* = 53 mothers Age of mothers: Average age was 35.0 ± 5.8 years Fathers: *n* = 45 fathers Age of fathers: Average age was 32.0 ± 4.6 years. Employment of parents: NR	Education of parents: NR Marital status: NR Family income: Average income was 429.39 JD (±116.399) about $605 ± $100 Setting: NR	To determine the parenting stress among parents of CYP with T1DM To find the factors creating stress among parents, and to identify the differential levels of stress in between fathers and mothers	Parenting stress index-short form (PSI-SF). The Arabic version Diabetes-specific PROM: no	(1) 83.7% of parents reported high stress levels, with 92.9% reporting parental distress (2) Mothers experienced higher levels of parental stress (*t* = −2.6, *p* < 0.05) than fathers (3) The subscale for parenting distress indicated higher parental distress in mothers (*t* = −2.4, *p* < 0.05)

13	MirRashidi et al. [47] 2021 (Iran)	Cross-sectional (descriptive analytical) study	Study population: *n* = 96 parents Fathers: *n* = 18 fathers Mothers: *n* = 78 Age of parents: NR Employment of parents:*n* = 41 employed, *n* = 2 retired, *n* = 53 unemployed Education of parents: *n* = 39 degree, *n* = 36 diploma, *n* = 3 illiterate Marital status: NR Family income: NR Setting: Recruitment was through the census		To evaluate the quality of life (QoL) in parents of children with T1DM	The Iranian version of the Health-Related Quality of Life (HRQoL) Questionnaire Diabetes-specific PROM: no	(1) Parents had an impaired feeling of vivacity (*p*=0.009) which may be related to depressive symptoms (2) Sufficient income was found to have a significant impact on health status of child and parent (*p*=0.007) (3) Impaired social activities and emotional problems impacted the QoL of fathers (*p*=0.023) (4) Limited availability of healthcare treatment led to negative emotions and a reduced QoL (5) Insufficient income affected their ability to perform daily activities and mental health
14	Khallaf et al. [55] 2022 (Egypt)	Cross-sectional study	Study population: *n* = 104 mothers Age of mothers: 37.87 ± 7.2 Employment of parents: NR Education of mothers: 60% secondary Age of fathers: 43.15 ± 8.8 Education of fathers: 70% secondary Marital status: NR Family income: 53.7% monthly income >2,000 EGP Setting: Paediatric diabetes clinic at an educational hospital in Cairo		To determine the level of diabetes-specific knowledge, maternal diabetes-related stress, coping strategies, and to measure their effect on child's glycaemic control	(1) Maternal diabetes-specific knowledge questionnaire (DKQ-24) (2) Maternal diabetes stress questionnaire (3) Maternal coping strategies, responses to stress questionnaire (RS-Diabetes), PSI) Diabetes-specific PROMS: yes	(1) Mothers had a good diabetes-specific knowledge score. No association between maternal diabetes-specific stress level and child's HBA1c level (2) The most common adopted coping strategies: acceptance of their child's illness (71.6%), emotional arousal (69.3%), Avoidance (68.8%) (3) The least adopted coping strategies: Involuntary disengagement (inaction, cognitive inference) emotional numbing. (4) Higher maternal education helped decrease HbA1c levels (*p* = 0.043) home glucose monitoring also decreased HbA1c (*p*=0.001), cognitive restructure about the disease also decreased HbA1c (*p*=0.0043)

15	Povlsen and Ringsberg [57] 2008 (Egypt)	Qualitative study no specific type	Study population: *n* = 7 parents Age of parents: NR Employment of parents: NR Education of parents: NR Marital status: NR Family income: NR Setting: The education center Assistance to Young Diabetics (AYD) in Cairo, Egypt		To explore variation in how parents living as immigrants in Egypt had perceived learning to live with a child with diabetes	Semi-structured interviews	(1) Time after diagnosis was difficult, tiring and demanding. (2) Mothers took on the main caring role (3) Worry about acute and long-term complications of the condition (4) Worry about CYP's ability to work and marry (5) Faith and hope were a common method of coping for parents

16	Oskouie et al. [49] 2013 (Iran)	Grounded theory	Study population: *n* = 15 parents *n* = 7 mothers *n* = 8 fathers Age of parents: NR Employment of parents: NR Education of parents: NR Marital status: NR Family income: NR Setting: The Diabetes Center of Shahroud University of Medical Sciences and the Gabric Diabetes Association in Tehran		To identify mediating factors affecting Iranian parents' coping processes with their children's T1DM	Semi-structured interviews	(1) Child's cooperation had a positive impact on parents' coping (2) The time of diagnosis was challenging (3) Parents found developmental and growth phases challenging (4) Economic factors were of concern to parents (5) Parents worried about their child's glycaemic control and their diabetes management skills (6) There is a need for increased psychological support from healthcare professionals and a need for economic support

17	Elissa et al. [4] 2017 (Palestine)	Qualitative descriptive design and content analysis	Study population: *n* = 10 parents *n* = 6 mothers, *n* = 4 fathers Age of mothers: Average age 28–49 years. Employment of mothers: All mothers were unemployed Education of mothers: *n* = 4 high school education *n* = 2 college education Age of fathers: Average 32-42 years	Employment of fathers: All fathers were employed Education of fathers: *n* = 3 high school, *n* = 1 college Marital status: NR Family income: NR Setting: Diabetes clinics in West Bank Palestine	To explore the experiences of daily life in CYP with T1DM and their parents living in the Palestinian West Bank	Semi-structured interviews	(1) Worry and concern for daughter's future and impact of condition on prospect of marriage and fertility (2) School stigmatization and discrimination against their CYP (3) Societal blame for giving birth to a CYP with diabetes, thus they hid their CYP's condition (4) Financial burden of CYP's treatment
18	Khandan et al. [45, 46] 2018 (Iran)	Descriptive phenomenological research	Study population: *n* = 11 mothers Age of Mothers: Mothers' age ranged from 30 to 48 years Employment of mothers: *n* = 5 employed, *n* = 6 unemployed Education of mothers *n* = 8 higher education, *n* = 3 high school education	Marital status: All mothers were married except one was divorced Family income: NR Setting: The study took place at the diabetes center in Kerman Iran	To explore the experiences of mothers with children with T1DM after the transfer of the caring role from hospital to home	Semi-structured interviews	(1) Caring for their child after the diagnosis was challenging (2) The financial burden of the condition was challenging for mothers (3) Mothers worried about their child's future (4) There was a lack of preparation or empowerment with knowledge and skills for diabetes management upon discharge

19	Khandan et al. 2018 [45, 46] (Iran)	Qualitative content analysis	Study population: *n* = 15 mothers Age of mothers: Mothers' age ranged from 25 to 48 years Employment of mothers: *n* = 7 employed, n = 8 unemployed Education of mothers: *n* = 3 no higher education *n* = 12 higher education	Marital status: *n* = 13 married *n* = 2 separated or divorced Family income: NR Setting: The diabetes centers in Kerman, Iran	To explore mothers' experiences in the maze path to the diagnosis of their children with T1DM	Semi-structured interviews	(1) Time of diagnosis was challenging (misdiagnosis, delayed diagnosis, complications) (2) Mothers suffered social isolation, societal blame, and depression after the diagnosis of their child (3) Cause of their child's condition was associated with a “divine exam” or an “inevitable destiny” therefore leading to acceptance (4) There is a need for increased psychological support from the healthcare professionals

20	Rossiter et al. [61] 2019 (United Arab Emirates)	Case-based descriptive qualitative design	Study population: *n* = 4 mothers Age of mothers: NR Employment of mothers: NR Education of mothers: All mothers had a college education Marital status: NR Family income: NR Family history: *n* = 3 family history of type 2 diabetes (T2D) *n* = 1 family history of T1DM Nationality of mothers: *n* = 1 UAE, *n* = Qatar, *n* = 1 Palestine, *n* = 1 Egypt Setting: Sheikh Khalifa Medical City (SKMC) a health service provider in Abu Dhabi		(1) To elicit an understanding of UAE mothers' perspectives and experiences of having a CYP diagnosed with T1DM. (2) To identify the role of family, community, culture, and healthcare team after diagnosis	Semi-structured interviews	(1) Mothers experienced feelings of shock, during the period of diagnosis (2) Family support and faith played a role in coping (3) Worry about their child's ability to marry and being judged by society (4) Psychological distress at the time of diagnosis was evident in the findings, increased support from healthcare professionals, the school and family enhanced the mothers' well-being

21	Asaad et al. 2022 [3] (Saudi Arabia)	Descriptive phenomenology inquiry design	Study population: *n* = 11 mothers Age of mothers: Average age 27–49 years Employment of mothers: NR Education of mothers: NR Age of fathers: NR Marital status: NR Family income: NR Setting: security forces hospital in Riyadh		To explore the experiences of Saudi mothers with children with T1DM and to understand their coping strategies and needs at time of diagnosis	Semi-structured interviews	(1) Mothers attributed the cause of their child's condition to concepts that stemmed from cultural contexts such as the “evil eye” (2) There is a need for better school support and the provision of school nurses (3) There is a need for culturally sensitive psychological support for parents with a second child with the condition (4) Participants who were “close to God” did not need traditional psychological support (5) There is a need to assess for diabetes distress in parents with more than one child with T1DM
22	Moghadam et al. [48] 2022 (Iran)	Qualitative study using content analysis	Study population: *n* = 20 mothers Age of mothers: Average age: 36.3 years Employment of mothers: NR Education of mothers: NR Marital status: NR	Family income: NR Setting: The Pediatric Hospital of Urmia, Iran	To explore the experience of mothers who had a child with T1DM from receiving the diagnosis and caring for their child	Semi-structured interviews	(1) Mothers identified personalized methods of coping due to burden of care (2) Social support helped ease the burden of care (3) Worry and fear of complications, and the constant need for care decreased the mother's QoL (4) Need for support from healthcare professionals and family members at time of diagnosis (5) Economic impact added to the burden of the disease

23	Momani et al. [58] 2022 (Jordan)	Constructive grounded theory design	Study population: *n* = 19 parents Age of parents: NR Employment of parents: NR	Education of parents: NR Marital status: NR Family income: NR Setting: NR	To understand how adolescents and their parents manage T1DM in Jordan. Only data related to the parents were included in this review	Semi-structured interviews	(1) Shock at diagnosis of their child with a chronic illness at such a young age (2) Acceptance was associated with the belief that it was their child's destiny to have the condition (3) Accepting “God's will” also helped with adaptation (4) Diabetes management was influenced by the collective nature of the Jordanian culture and was underpinned by religious beliefs

**Table 2:** List of PROMs included in review.

Name of PROM	Number of studies that included PROM	Use of PROM
Self-efficacy for diabetes scale parent version (SED-P)	*n* = 1 [42]	To measure self-efficacy related to diabetes (diabetes specific)
Hypoglycemia fear survey parenting version (HFS-P)	*n* = 1 [42]	To measure fear of hypoglycaemia (diabetes specific)
Parenting stress index (PSI)	*n* = 2 [50, 59]	To assess parental distress
Health-related quality of life (HRQoL)	*n* = 2 [47, 52]	To assess emotional and social well-being
Self-developed tools	*n* = 2 [60, 62]	To assess psychological distress and worry
Rahim anxiety depression (RAD)	*n* = 1 [50]	To assess insomnia
Peds quality of life family impact module (Peds QL FIM)	*n* = 1 [52]	To assess worry and emotional distress
Pediatric inventory for parents (PIP)	*n* = 1 [53]	To assess emotional distress
Depression anxiety stress scale (DASS)	*n* = 1 [50]	To assess anxiety
Symptom checklist-90 (SCL-90)	*n* = 1 [51]	To assess anxiety
Health promoting lifestyle profile (HPLPII)	*n* = 1 [43]	To assess psychological health
Diabetes knowledge questionnaire-24 (DKQ-24)	*n* = 1 [55]	To assess diabetes-related knowledge (diabetes specific)
Responses to stress questionnaire (RSQ). The diabetes domain of the measure	*n* = 1 [55]	To measure diabetes-related stress and voluntary coping

**Table 3:** Psychometric properties of included PROMs.

Psychometric property	Number of studies that assessed it	Findings
Content validity	*n* = 3 [43, 44, 59]	(1) One study had “adequate” COSMIN quality rating as they provided evidence to support content validity, they had 10 experts check the PROM for the content validity ratio (CVR) which was found to be >0.62 [43] (2) Two studies had a “doubtful” COSMIN quality rating as they report that it was checked in previous studies as there was insufficient information in the study to indicate if a panel of experts was involved in the content validity process [44, 59]

Internal consistency	*n* = 11 [42, 44, 47, 50, 51, 52, 53, 54, 55, 56, 60]	(1) Most studies used the measurement of Cronbach's alpha coefficient which has been found to be an appropriate method for the measurement of internal consistency [38]. The values of the Cronbach's alpha in these studies ranged from (*α* = 0.96–0.74), a value of around 0.70 or greater is widely considered desirable [63] (2) Eight studies had an “adequate” COSMIN quality rating for the level of internal consistency [42, 44, 47, 53, 54, 55, 56, 59] (3) Three studies had a “low” COSMIN quality rating; thus, their reliability can be considered questionable [50, 51, 60]

Reliability (test–retest)	*n* = 4 [42, 43, 52, 56]	(1) One study demonstrated an intraclass correlation coefficient (ICC) of 0.90 indicating good reliability [43], as values range between 0 and 1 with values closer to 1 indicating stronger reliability [64] (2) Three studies pilot tested the PROM on 10 participants [43, 52, 56] (3) One study mentioned that the PROM was checked for reliability but no method or data were reported [54] (4) Three studies had a “adequate” COSMIN quality rating for reliability [43, 52, 56]

Hypothesis testing for construct validity	Most studies did not report construct validity, or it was difficult to determine if they did	(1) One study reported that the content validity index (CVI) was assessed no value was reported [43] (2) Forward–backward translation was reported in four studies [43, 52, 53, 55]; however, it requires cognitive testing to compare these tests against the original so there is more confidence that the adapted instrument is measuring a construct comparable to the original [65]. This was not done in any of the included studies

Other psychometric properties	*n* = 0	(1) None of the studies were found to demonstrate cross-cultural validity/measurement invariance, measurement error, criterion validity, or responsiveness

## Data Availability

All data are included in supplementary material tables.

## Supplementary Materials

Supplementary Materials Additional supplementary materials with supporting information may be found here.
