# Supplementary material for: Psychosocial–Spiritual Experiences and Outcomes in Parents of Children with Type 1 Diabetes Mellitus from the Middle East and North Africa Region: A Systematic Review
Source: Pediatr Diabetes. 2024 Jul 12;2024:6111661. doi: 10.1155/2024/6111661 (PMC12016858; doi:10.1155/2024/6111661)
Supplement: Supplementary Materials — Additional supplementary materials with supporting information may be found here. [file 6111661.f1.docx]

Supplementary files:

Supplementary Table 1 Facet analysis using PEO search approach for review questions

| **Search approach of review questions** | | | |
| --- | --- | --- | --- |
| **Key concepts** | **Population** | **Exposure** | **Outcome** |
|  | Parents of CYP with T1DM in the MENA region. | Child’s diagnosis with T1DM | Psychosocial outcomes |
| **Search terms** | Parent* OR, Mother* OR Matern*, Father* OR Patern*, OR Famil* adj2 member* | AND  Type 1 diabetes mellitus OR T1DMM OR Type 1 diabetes OR T1DM OR Insulin dependent diabetes mellitus OR Juvenile onset diabetes mellitus OR Insulin depend* diabet* OR IDDM OR Diabetes mellitus type 1 OR Diabetes type 1 | AND  Diabetes adj3 distress OR Diabetes relate* distress OR Psycholog* distress OR Psycholog*adj3 stress OR Psycholog* factor* OR Psychosocial adj2 distress OR Psychosocial adj2 stress OR Emotion* adj3 stress OR Emotion* adj3 distress OR Emotional factor* OR Psycholog* wellbeing OR Psycholog* well-being OR Experience* OR Emotional experience* OR Quality of life |
| **Limits** | English and Arabic related studies |  |  |

Supplementary Table 2 Summary of PROMS used in quantitative studies

| **Name of scale** | **Number of studies that used scale** | **Purpose of scale** | **Subscales of scale** | | **Psychosocial constructs of scale** |
| --- | --- | --- | --- | --- | --- |
| Parenting Stress Index-Short Form (PSI-SF). The Arabic version. | 2  (46, 56) | - Is a 36-item measure of parental stress - Likert-style rating 1-5 (strongly disagree-strongly agree) - Each subscale has 12 items. | Parental Distress (PD)  Parent-Child Dysfunctional Interaction (PCDI)  Difficult Child (DC) | | Parental distress |
| Pediatric Inventory for Parents (PIP). | 1  (51) | - Is a 42-item measure of the levels of stress suffered by parents caring for a child with a chronic disease. - Likert-style rating 1-5 (never-very often - It has 4 subscales | Communication (CM)  Emotional Distress (ED)  Medical Care (MC)  Role Function (RF) | | Emotional distress |
| Self-Efficacy for Diabetes Scale Parent version (SED-P) | 1  (40) | - Is a 35-item specific measure of diabetes-specific self-efficacy - Likert style rating 1-6 (“very sure I can’t”- “very sure I can”) - It has 3 subscales | Diabetes-specific self-efficacy (SED-D)  Medical self-efficacy (SED-M)  General self-efficacy (SED-G) | | Diabetes-specific self-efficacy  General self-efficacy |
| Hypoglycaemia Fear Survey Parent version (HFS-P) | 1  (40) | - Is a 26-item measure of parent’s fear of hypoglycaemia in their children - Likert style rating 1-5 (never-very often) - It has 2 subscales | Behaviour  Worry | | Worry |
| Ways of Coping Questionnaire (WCQ) | 1  (53) | - Is a 66-item measure of coping processes, to assess thoughts and acts used to deal with stressful encounters. - Likert-style rating 1-4 (not used/not applicable-used a great deal). - It has 8 coping subscales | Confrontive coping  Distancing  Self-controlling  Seeking social support | Accepting responsibility  Escape, Avoidance  Planful problem-solving  Positive reappraisal | Confrontive coping  Seeking social support  Positive reappraisal  Accepting responsibility  Self-control |
| The Diabetes Knowledge Questionnaire-24 (DKQ-24) | 1  (53, 55) | - Is a 24-item measure for assessing understanding of diabetes knowledge. - Likert style rating (yes, no, I don’t know) - Does not have subscales. | Not reported | | Not reported |
| Health-Related Quality of Life (HRQoL) Persian version and Arabic version | 2  (45, 52) | - Is a measure used to examine the impact of disease on quality of life - It has 4 subscales | Physical  Functional  Emotional  Social well-being | | Emotional  Social well-being |
| Depression Anxiety Stress Scale (DASS) | 1  (46) | - Is a 42-item measure of depression, anxiety, and stress. - Likert-style rating 0-4 (“Does not apply to me”- “applied to me very much/most of the time”. - Does not have subscales | Not reported | | Anxiety  Stress |
| Responses to Stress Questionnaire (RSQ) | 1  (55) | - Is a 57 -item measure of coping with specific domains of stress - Likert-style rating 1-4. - Has a diabetes domain | 3 diabetes-related subscales | | Coping  Stress  Worry |
| Symptom Checklist-90 (SCL-90) | 1  (44) | - It is a 90-item measure to assess psychological problems. - Likert-style rating 0-4 (not at all-extremely) - It has 9 subscales | Somatization  Obsessive-compulsive  Interpersonal sensibility  Depression  Anxiety | Anger-hostility  Phobic anxiety  Paranoid ideation  Psychoticism | Somatization  Interpersonal sensibility  Depression  Anxiety  Anger-hostility  Phobic anxiety |
| Family Quality of Life scale was used (Persian version). | 1  (41) | - Is a 25-item measure to assess family’s perceived perception of quality of life. - Likert-style rating 1-5 (very dissatisfied- very satisfied) - It has 5 subscales. | Family interaction  Parenting  Emotional well-being,  Physical/material well-being  Disability-related supports | | Emotional well-being  Family interaction |
| Rahim Anxiety Depression (RAD) questionnaire. | 1  (50) | - Is a measure of screening for minor psychiatric conditions. - Likert-style rating 0-3 (no, mild, moderate, severe). - It has 7 subscales | Nervousness  Irritability  Insomnia  Fatigue | Poor concentration  Fear  Indecision | Nervousness  Irritability  Insomnia Fatigue  Poor concentration  Fear  Indecision |
| Peds Quality of Life Family Impact Module (Peds QL FIM) | 1  (52) | - Is a 36-item measure of the impact of the child’s chronic health condition of the parents and family. - It has 8 subscales. | Physical functioning  Emotional functioning  Social functioning  Cognitive functioning | Communication  Worry  Daily activities  Family relationships | Emotional functioning  Social functioning  Worry |
| Health Promoting Lifestyle Profile (HPLPII) | 1  (47) | - Is a 52-item measure to determine how engaged people are with health promoting behaviours. - Likert-style rating 1-4 (never, sometimes, often, routinely) - It has 3 subscales | Health responsibility (HR)  Spiritual growth (SG)  Physical and psychological health | | Psychological health |

Supplementary Table 3 Psychosocial outcomes and constructs of all included studies

| **Psychosocial constructs reported in study** | | | | Anxiety | Emotional/  Psychological well-being | | | Quality of Life (QoL) | Emotional /psychological /social distress | | Stress | Worry | | Burden of care | | Fear | | Self-efficacy/control | | | Coping |
| --- | --- | --- | --- | --- | --- | --- | --- | --- | --- | --- | --- | --- | --- | --- | --- | --- | --- | --- | --- | --- | --- |
| 1 | AlBuhairan *et al.* 2016 (Quant) | | |  | √ | | | √ |  | |  | √  Complications | |  | |  | |  | | |  |
| 2 | Aldubayee *et al.* 2020 (Quant) | | |  |  | | |  |  | | √ |  | |  | |  | |  | | |  |
| 3 | Amiri *et al.* 2018 Quant) | | |  |  | | |  | √ | | √ |  | |  | | √  hypoglycaemia | | √ | | |  |
| 4 | Asaad *et al.* 2022 (Qual) | | |  | √ | | |  | √  psychological | |  |  | |  | |  | |  | | | √  Faith |
| 5 | Asadi-Shavaki *et al.* 2020 (Quant) | | |  |  | | |  |  | | √ |  | |  | |  | |  | | | √ |
| 6 | Elissa *et al.* 2017 (Qual) | | |  |  | | |  |  | |  | √  Financial/ Marriage | |  | |  | |  | | |  |
| 7 | Felimban *et al.* 1998 (Quant) | | |  |  | | |  | √  psychological | |  |  | |  | | √ | |  | | |  |
| 8 | Hashemipour-Zavareh *et al.* 2020 (Quant) | | |  |  | | | √ |  | |  |  | |  | |  | |  | | |  |
| 9 | Khallaf *et al.* 2022 (Quant) | | |  |  | | |  |  | | √ | √ | |  | |  | |  | | | √ |
| 10 | Khandan *et al.* 2018 a (Qual) | | |  | √ | | |  | √  Psychological  Social | |  |  | |  | |  | |  | | | √  Faith |
| 11 | Khandan *et al.* 2018 b (Qual) | | |  |  | | |  | √  Psychological  Social | |  | √  Financial/ Marriage/complications | |  | |  | |  | | |  |
| 12 | Mahfouz *et al.* 2018 (Quant) | | |  |  | | |  | √  Social | |  | √  Marriage | |  | |  | | √ | | | √ |
| **Psychosocial constructs reported in study** | | Anxiety | Emotional/  Psychological well-being | | | Quality of Life (QoL) | Emotional/  Psychological/ social distress | | | Stress | Worry | | Burden of care | | Fear | | Self-efficacy/  Control | | Coping |  |  |
| 13 | MirRashidi *et al.* 2021 (Quant) |  | √ | | | √ |  | | |  | √  Financial | |  | |  | |  | |  |  |  |
| 14 | Moghadam *et al.* 2022 (Qual) |  |  | | | √ | √ | | |  | √  Marriage/complications | | √ | |  | |  | | √ |  |  |
| 15 | Momani *et al.* 2022 (Qual) |  | √ | | |  | √ | | |  |  | |  | |  | |  | | √  Faith |  |  |
| 16 | Noueiri & Nassif 2018 (Quant) | √  _separation_ |  | | |  |  | | |  | √  Financial/Work | |  | |  | |  | |  |  |  |
| 17 | Obaid *et al.* 2020 (Quant) | √ |  | | |  | √ | | |  | √  Financial | | √ | |  | |  | |  |  |  |
| 18 | Obeidat *et al*. 2020 (Quant) |  |  | | |  | √ | | | √ |  | |  | |  | |  | |  |  |  |
| 19 | Oskouie *et al.* 2013 (Qual) |  | √ | | |  |  | | |  | √  Financial | |  | |  | |  | | √ |  |  |
| 20 | Povlsen & Ringsberg 2008 (Qual) |  |  | | |  | √ | | |  | √  Marriage/complications | |  | |  | |  | | √  Faith |  |  |
| 21 | Rossiter *et al.* 2019 (Qual) |  | √ | | |  | √ | | |  | √  Marriage | |  | |  | |  | |  |  |  |
| 22 | Saghaei *et al.* 2017 (Quant) | √ |  | | |  |  | | | √ |  | |  | |  | |  | | √ |  |  |
| 23 | Talakoub & Nassiri 2019 (Quant) | √ | √ | | |  |  | | |  |  | |  | |  | |  | |  |  |  |

Supplementary Table 4 Summary of psychometric properties of PROMS using the COSMIN checklist

| **PROM** | **Content Validity** | | **Structural validity** | | **Internal consistency** | | **Cross-cultural validity/ measurement invariance** | | **Reliability** | | **Hypothesis testing for construct validity** | |
| --- | --- | --- | --- | --- | --- | --- | --- | --- | --- | --- | --- | --- |
|  | Rating of results | Quality of evidence | Rating of results | Quality of evidence | Rating of results | Quality of evidence | Rating of results | Quality of evidence | Rating of results | Quality of evidence | Rating of results | Quality of evidence |
| **PSI-SF-Arabic**  Parenting Stress Index-Short Form, Arabic version | ?(They mention it was checked in previous studies) | Moderate | ? | Moderate | + (Cronbach’s alpha coefficient was 0.91 for the total translated Arabic scale) | Moderate | NR | NR | NR | NR | NR | NR |
| **PSI-SF-Persian**  Parenting Stress Index-Short Form, Persian version | NR | NR | NR | NR | NR | NR | NR | NR | NR | NR | NR | NR |
| **PIP-Arabic**  Pediatric Inventory for Parents, Arabic version | ? | Moderate | NR | NR | + (Cronbach’s alpha coefficient for both scales frequency: α = 0.94, difficulty: α = 0.96) | Moderate | NR | NR | NR | NR | + forward (into Arabic) backward translation | Low |
| **PIP-Persian**  Pediatric Inventory for Parents, Persian version | ? | Low | NR | NR | + (Cronbach’s alpha coefficient of 0.93 for total translated Persian scale) | Moderate | NR | NR | ± (test-retest was done) | Low | ? | Low |
| **SED-Persian**  Self-Efficacy for Diabetes Scale Parent version, Persian version | ? | Low | NR | NR | + (Cronbach’s alpha coefficient of 0.74 in both mothers and fathers). | Moderate | NR | NR | ± (test-retest was done) | Low | ? | Low |
| **HFS-Persian**  Hypoglycaemia Fear Survey Parent version, Persian version | ? | Low | NR | NR | + (Cronbach’s alpha coefficient for total scale: mothers 0.94; fathers 0.94). | Moderate | NR | NR | ± (test-retest was done) | Low | ? | Low |
| **RSQ-Diabetes domain** Responses to Stress Questionnaire | ? | ? | NR | NR | + (Cronbach’s alpha coefficient for part I 0.82, for part II 0.89) | Moderate | NR | NR | + (pilot study was done) | Low | + forward (into Arabic) backward translation | Low |
| **WCQ-Arabic**  Ways of Coping Questionnaire, Arabic version | ? | Moderate | NR | NR | + (Cronbach’s alpha coefficient for the scale was 0.91) | Moderate | NR | NR | + (pilot study and test-retest was done) | Moderate | ± | Moderate |
| **DKQ-24-Arabic**  The Diabetes Knowledge Questionnaire-24, Arabic version | ? | Moderate | NR | NR | + (Cronbach’s alpha for the scale was 0.82) | Moderate | NR | NR | + (pilot study and test-retest was done) | Moderate | ± | Moderate |
| **HRQoL-Persian**  Health-Related Quality of Life, Persian version. | ? | Low | NR | NR | + (Cronbach’s alpha coefficients ranged from 0.77 to 0.90 except for the vitality scale (α=0.65). | Moderate | NR | NR | ? | Low | ? | Low |
| **DASS-Persian**  Depression Anxiety Stress Scale, Persian version | NR | NR | NR | NR | NR | NR | NR | NR | NR | NR | NR | NR |
| **SCL-90-Persian**  Symptom Checklist-90, Persian version | NR | NR | NR | NR | NR | NR | NR | NR | NR | NR | NR | NR |
| **FQoL-Persian**  Family Quality of Life, Persian version | ? (They mention it was checked in previous studies) | Low | NR | NR | + Cronbach’s alpha coefficient of the scale for total quality of life was 0.983 | Low | NR | NR | NR | NR | NR | NR |
| **RAD-Arabic**  Rahim Anxiety Depression, Arabic version | ? | Low | NR | NR | + Guttman split-half method was used to test the reliability of the scale and was found to be about 87 | Low | NR | NR | NR | NR | NR | NR |
| **Peds QL-FIM-Arabic** Peds Quality of Life Family Impact Module, Arabic version | ? | Low | NR | NR | ± Cohen’s kappa coefficient was used but no value was shared | Low | NR | NR | + test-retest piloted on 10 participants | Moderate | + forward (into Arabic) backward translation | Moderate |
| **HPLPII-Persian**  Health Promoting Lifestyle Profile, Persian version | + (10 expert faculty members assessed content validity ratio (CVR) >0.62. ) | Moderate | NR | NR | ± they mention they checked for reliability and validity but didn’t share methods or data. | Low | NR | NR | + test-retest ICC of 0.90 piloted on 10 participants | Moderate | ± they mention that content validity index was assessed but did not provide details.  + forward (into Persian) backward translation | Low |
| **Self-developed PROM (Noueiri & Nassif 2018)** related to psychological and financial impact of T1DM and its oral complications on the families. | ? | Very low | NR | NR | NR | NR | NR | NR | NR | NR | NR | NR |
| **Self-developed PROM (Obaid 2020)** to measure burden (psychological, social, and economical) of caring for children with T1DM upon mothers. | ? was checked with experts | Very low | NR | NR | NR | NR | NR | NR | ? PROM was developed with mothers, but methods of development not shared | Very low | NR | NR |
| **Self-developed section of PROM (Asadi-Shavaki 2020)** related to the constructs of the transactional model, | ? (Developed by the research team with confirmed validity and reliability) | Low | NR | NR | NR | NR | NR | NR | ? They mention reliability was checked but do not include the method | Low | NR | NR |

*± inconsistent results; - unsatisfactory results; +satisfactory results; NR not reported; ?unclear

Supplementary Table 5 Quality assessment of included cross-sectional studies (AXIS tool for cross-sectional studies)

| Question 1 | | Question 2 | Question 3 | Question 4 | Question 5 | Question 6 | Question 7 | Question 8 | Question 9 | Question 10 | Question 11 | Question 12 | Question 13 | Question 14 | Question 15 | Question 16 | Question 17 | Question 18 | Question 19 | Question 20 |
| --- | --- | --- | --- | --- | --- | --- | --- | --- | --- | --- | --- | --- | --- | --- | --- | --- | --- | --- | --- | --- |
|  | Were the aims clear? | Was the study design appropriate for the stated aim(s)? | Was the sample size justified? | Was the target/reference population clearly defined? (Is it clear who the  research was about?) | Was the sample frame taken from an appropriate population base so that it  closely represented the target/reference population under investigation? | Was the selection process likely to select subjects/participants that were  representative of the target/reference population under investigation? | Were measures undertaken to address and categorise non-responders? | Were the risk factor and outcome variables measured appropriate to the aims  of the study? | Were the risk factors  and outcome variables measured correctly  using  instruments/measurements that had been  trialed, piloted or published  previously? | Is it clear what was used to determined statistical significance and/or  precision estimates? (e.g. p-values, confidence intervals) | Were the methods (including statistical methods) sufficiently described to  enable them to be repeated? | Were the basic data adequately described? | Does the response rate raise concerns about non-response bias? | If appropriate, was information about non-responders described? | Were the results internally consistent? | Were the results presented for all the analyses described in the methods? | Were the authors' discussions and conclusions justified by the results? | Were the limitations of the study discussed? | Were there any funding sources or conflicts of interest that may affect the  authors’ interpretation of the results? | Was ethical approval or consent of participants attained? |
| Author | | | | | | | | | | | | | | | | | | | | |
| AlBuhairan *et al.* 2016 | | | | | | | | | | | | | | | | | | | | |
| VT | Yes | Yes | No | Yes | Don’t know/can’t tell | Yes | No | Yes | Yes | Yes | No | Yes | Don’t know/can’t tell | No | Don’t know/can’t tell | Yes | Yes | No | No | Yes |
| MA | Yes | Yes | No | Yes | Don’t know/can’t tell | Yes | No | Yes | Yes | Yes | No | Yes | Don’t know/can’t tell | No | Don’t know/can’t tell | Yes | Yes | No | No | Yes |
| AlDubayee *et al.* 2020 | | | | | | | | | | | | | | | | | | | | |
| VT | Yes | Yes | Yes | Yes | Yes | Yes | Yes | Yes | Yes | Yes | Yes | Yes | Don’t know/can’t tell | No | Yes | Yes | Yes | No | No |  |
| MA | Yes | Yes | Yes | Yes | Yes | Yes | Yes | Yes | Yes | Yes | Yes | Yes | Don’t know/can’t tell | No | Yes | Yes | Yes | No | No |  |
| Amiri *et al.* 2018 | | | | | | | | | | | | | | | | | | | | |
| VT | Yes | Yes | Yes | Yes | Yes | Yes | No | Yes | Yes | Yes | No | Yes | Don’t know/can’t tell | No | Yes | Yes | Yes | Yes | No | Yes |
| MA | Yes | Yes | Yes | Yes | Yes | Yes | No | Yes | Yes | Yes | No | Yes | Don’t know/can’t tell | No | Yes | Yes | Yes | Yes | No | Yes |
| Asadi-Shavaki *et al.* 2020 | | | | | | | | | | | | | | | | | | | | |
| VT | Yes | Yes | Yes | Yes | Don’t know/can’t tell | Don’t know/can’t tell | No | Yes | Yes | Yes | Don’t know/can’t tell | Yes | Don’t know/can’t tell | No | Yes | Yes | Yes | Don’t know/can’t tell | No | Yes |
| MA | Yes | Yes | Yes | Yes | Don’t know/can’t tell | Don’t know/can’t tell | No | Yes | Yes | Yes | Don’t know/can’t tell | Yes | Don’t know/can’t tell | No | Yes | Yes | Yes | Don’t know/can’t tell | No | Yes |
| Khallaf *et al.* 2022 | | | | | | | | | | | | | | | | | | | | |
| VT | Yes | Yes | Yes | Yes | Don’t know/can’t tell | Don’t know/can’t tell | Yes/No (address non responders: Yes categorise: No | Don’t know/can’t tell | Yes | Yes | Yes | Yes | Don’t know/can’t tell | No | Don’t know/can’t tell | Yes | Yes | Yes | No | Yes |
| MA | Yes | Yes | Yes | Yes | Don’t know/can’t tell | Don’t know/can’t tell | Yes/No (address non responders: Yes categorise: No | Don’t know/can’t tell | Yes | Yes | Yes | Yes | Don’t know/can’t tell | No | Don’t know/can’t tell | Yes | Yes | Yes | No | Yes |
| Mahfouz *et al.* 2018 | | | | | | | | | | | | | | | | | | | | |
| VT | Yes | Yes | No | Yes | Yes | Yes | No (but non-responders are mentioned) | Yes | Yes | Yes | No | Yes | Don’t know/can’t tell | No | Yes | Yes | Yes | Yes | No | Yes |
| MA | Yes | Yes | No | Yes | Yes | Yes | No | Yes | Yes | Yes | No | Yes | Don’t know/can’t tell | No | Yes | Yes | Yes | Yes | No | Yes |
| MirRashidi *et al.* 2018 | | | | | | | | | | | | | | | | | | | | |
| VT | Yes | Yes | No | Yes | Don’t know/can’t tell | Yes | No | Yes | Yes | No (Statistical Analysis method not reported) | No | Yes | Don’t know/can’t tell | No | Yes | No | Yes | No | No | Yes |
| MA | Yes | Yes | No | Yes | Don’t know/can’t tell | Yes | No | Yes | Yes | No(Statistical Analysis method not reported) | No | Yes | Don’t know/can’t tell | No | Yes | No | Yes | No | No | Yes |
| Noueiri & Nassif 2018 | | | | | | | | | | | | | | | | | | | | |
| VT | Yes | Yes | No | Yes | Don’t know/can’t tell | Don’t know/can’t tell | No | Yes | No | Yes | No | Yes | Don’t know/can’t tell | No | Don’t know/can’t tell | Yes | Yes | No | Don’t know/can’t tell | Yes |
| MA | Yes | Yes | No | Yes | Don’t know/can’t tell | Don’t know/can’t tell | No | Yes | No | Yes | No | Yes | Don’t know/can’t tell | No | Don’t know/can’t tell | Yes | Yes | No | Don’t know/can’t tell | Yes |
| Obaid *et al.* 2020 | | | | | | | | | | | | | | | | | | | | |
| VT | No (not reported in intro, but reported in methods) | Don’t know/can’t tell | No | No | Don’t know/can’t tell | Don’t know/can’t tell | No | Don’t know/can’t tell | Don’t know/can’t tell | No (Statistical Analysis method not reported) | No | Yes | Don’t know/can’t tell | No | No | Yes | Yes | No | No | No |
| MA | No (Not reporter) | Don’t know/can’t tell | No | No | Don’t know/can’t tell | Don’t know/can’t tell | No | Don’t know/can’t tell | Don’t know/can’t tell | No(Statistical Analysis method not reported) | No | Yes | Don’t know/can’t tell | No | No | Yes | Yes | No | No | No |
| Obeidat *et al.* 2020 | | | | | | | | | | | | | | | | | | | | |
| VT | Don’t know/can’t tell | Don’t know/can’t tell | Yes | Yes | Don’t know/can’t tell | Yes | No | Yes | Yes | Yes | Yes | Yes | Don’t know/can’t tell | No | Yes | Yes | Yes | No | No | Yes |
| MA | Don’t know/can’t tell | Don’t know/can’t tell | Yes | Yes | Don’t know/can’t tell | Yes | No | Yes | Yes | Yes | Yes | Yes | Don’t know/can’t tell | No | Yes | Yes | Yes | No | No | Yes |
| Talakoub & Nasiri 2012 | | | | | | | | | | | | | | | | | | | | |
| VT | No | Don't know/Can't tell | No | Yes | Don't know/Can't tell | Don't know/Can't tell | No | Don't know/Can't tell | Don't know/Can't tell | No | No | Yes | Don't know/Can't tell | No | No | Yes | Yes | No | Don't know/Can't tell | No |
| MA | No | Don't know/Can't tell | No | Yes | Don't know/Can't tell | Don't know/Can't tell | No | Don't know/Can't tell | Don't know/Can't tell | No | No | Yes | Don't know/Can't tell | No | No | Yes | Yes | No | Don't know/Can't tell | No |

Supplementary Table 6 Quality assessment of included case-studies

| Question 1 | | Question 2 | Question 3 | Question 4 | Question 5 | Question 6 | Question 7 | Question 8 | Question 9 | Question 10 |
| --- | --- | --- | --- | --- | --- | --- | --- | --- | --- | --- |
|  | Were the groups comparable other than the presence of disease in cases or the absence of disease in controls? | Were cases and controls matched appropriately? | Were the same criteria used for identification of cases and controls? | Was exposure measured in a standard, valid and reliable way? | Was exposure measured in the same way for cases and controls? | Were confounding factors identified? | Were strategies to deal with confounding factors stated? | Were outcomes assessed in a standard, valid and reliable way for cases and controls? | Was the exposure period of interest long enough to be meaningful? | Was appropriate statistical analysis used? |
| **Authors** | | | | | | | | | | |
| **Felimban *et al.* 1998** | | | | | | | | | | |
| VT | Yes | Yes | Yes | Yes | Yes | No | No | Yes | Unclear | Yes |
| MA | Yes | Yes | Yes | Yes | Yes | No | No | Yes | Unclear | Yes |
| **Hashemipour-Zavareh *et al.* 2020** | | | | | | | | | | |
| VT | Yes | Yes | Yes | Yes | Yes | No | No | Yes | Unclear | Yes |
| MA | Yes | Yes | Yes | Yes | Yes | No | No | Yes | Unclear | Yes |

Supplementary Table 7 Quality assessment of included quasi-experimental studies

|  | Question 1 | Question 2 | Question 3 | Question 4 | Question 5 | Question 6 | Question 7 | Question 8 | Question 9 |
| --- | --- | --- | --- | --- | --- | --- | --- | --- | --- |
|  | Is it clear in the study what is the 'cause' and what is the 'effect'? | Were the participants included in any comparisons similar? | Were the participants included in any comparisons receiving similar treatment/care, other than the exposure or intervention of interest? | Was there a control group? | Were there multiple measurements of the outcome both pre and post the intervention/ exposure? | Was follow up complete and if not, were differences between groups in terms of their follow up adequately described and analysed? | Were the outcomes of participants included in any comparisons measure in the same way? | Were outcomes measured in a reliable way? | Were appropriate statistical analysis used? |
| **Authors** | | | | | | | | | |
| **Saghaei *et al.* 2017** | | | | | | | | | |
| VT | Yes | Yes | Unclear | Yes | Yes | Unclear | Yes | Yes | Yes |
| MA | Yes | Yes | Unclear | Yes | Yes | Unclear | Yes | Yes | Yes |

Supplementary Table 8 Quality assessment of included qualitative studies

|  | Question 1 | Question 2 | Question 3 | Question 4 | Question 5 | Question 6 | Question 7 | Question 8 | Question 9 | Question 10 |
| --- | --- | --- | --- | --- | --- | --- | --- | --- | --- | --- |
|  | Is there congruity between philosophical perspective and research methodology? | Is there congruity between research methodology and research question or objectives? | Is there congruity between research methodology and methods used to collect data? | Is there congruity between research methodology and representation and analysis of data? | Is there congruity between research methodology and interpretation of results? | Is there a statement locating the researcher culturally or theoretically? | Is the influence of the researcher on the research, and vice- versa, addressed? | Are participants, and their voices, adequately represented? | Is the research ethical according to current criteria or, for recent studies, and is there evidence of ethical approval by an appropriate body? | Do the conclusions drawn in the research report flow from the analysis, or interpretation, of the data? |
| **Author** | | | | | | | | | | |
| **Asaad *et al.*2022** | | | | | | | | | | |
| VT | Yes | Yes | Yes | Yes | Yes | Yes | Unclear | Yes | Yes | Yes |
| MA | Yes | Yes | Yes | Yes | Yes | Yes | Unclear | Yes | Yes | Yes |
| **Elissa *et al.* 2017** | | | | | | | | | | |
| VT | Yes | Yes | Yes | Yes | Yes | No | No | Yes | Yes | Yes |
| MA | Yes | Yes | Yes | Yes | Yes | No | No | Yes | Yes | Yes |
| **Khandan *et al.* (a)** | | | | | | | | | | |
| VT | Yes | Yes | Yes | Yes | Yes | No | No | Yes | Yes | Yes |
| MA | Yes | Yes | Yes | Yes | Yes | No | No | Yes | Yes | Yes |
| **Khandan *et al.* (b)** | | | | | | | | | | |
| VT | Unclear | Yes | Yes | Yes | Yes | No | No | Unclear | Yes | Yes |
| MA | Unclear (philosophical perspective not stated) | Yes | Yes | Yes | Yes | No | No | Unclear | Yes | Yes |
| **Moghadam *et al.* 2022** | | | | | | | | | | |
| VT | Yes | Yes | Yes | Yes | Yes | No | No | Yes | Yes | Yes |
| MA | Yes | Yes | Yes | Yes | Yes | No | No | Yes | Yes | Yes |
| **Momani *et al.* 2022** | | | | | | | | | | |
| VT | Unclear | Yes | Yes | Yes | Yes | No | No | Yes | Yes | Yes |
| MA | Unclear | Yes | Yes | Yes | Yes | No | No | Yes | Yes | Yes |
| **Oskouie *et al.* 2013** | | | | | | | | | | |
| VT | Yes | Yes | Yes | Yes | Yes | No | No | Yes | Yes | Yes |
| MA | Yes | Yes | Yes | Yes | Yes | No | No | Yes | Yes | Yes |
| **Povlsen & Ringsberg 2008** | | | | | | | | | | |
| VT | Yes | Yes | Yes | Yes | Yes | No | Unclear | Yes | Yes | Yes |
| MA | Yes | Yes | Yes | Yes | Yes | No | Unclear | Yes | Yes | Yes |
| **Rossiter *et al.* 2019** | | | | | | | | | | |
| VT | Yes | Yes | Yes | Yes | Yes | No | No | Unclear (small sample size) | Yes | Yes |
| MA | Yes | Yes | Yes | Yes | Yes | No | No | Unclear (n=5) | Yes | Yes |

Supplementary Table 9 GRADE assessment

| **GRADE** | | | | | | | | | |
| --- | --- | --- | --- | --- | --- | --- | --- | --- | --- |
| **Population:** Parents of children with T1DM | | | | | | | | | |
| **Setting:** MENA region | | | | | | | | | |
|  | **Study Contributing to outcome** | **Study design** | **Risk of Bias** | **Inconsistency** | **Indirectness** | **Imprecision** | **Publication Bias** | **Comments** | **Total quality Score** |
| **Outcomes** | | | | | | | | | |
| **Physical** | | | | | | | | | |
| Insomnia/sleep | Felimban *et al.* 1998, n=110 participants. Rahim Anxiety Depression (RAD) questionnaire | Case-control study (non-RCT start as **low**) | Low (No serious risk of bias. Cochrane risk of bias tool is not applicable – Lack of blinding, allocation concealment, loss of follow up not relevant)  Do not downgrade | Low (One study assessed this outcome, inconsistency is not an issue) Do not Downgrade | Low (only patients from the primary healthcare sector were included, this outcome addressed the review question, therefore no indirectness exists)  Do not Downgrade | Very low (study has a small sample size <400 therefore some imprecision exists). Downgrade -1 | Undetected Do not downgrade | Outcome only found in one study, and sample size affected imprecision of data Some confounding variables accounted for but not all. | Very Low |
| Fatigue | Felimban *et al.* 1998, n=110 participants. Rahim Anxiety Depression (RAD) questionnaire | Case-control study (non-RCT start as **low**) | Low (No serious risk of bias. Cochrane risk of bias tool is not applicable – Lack of blinding, allocation concealment, loss of follow up are not relevant)  Do not downgrade | Low (One study assessed this outcome, inconsistency is not an issue) Do not Downgrade | Low (only patients from the primary health are sector were included, this outcome addressed the review question, therefore no indirectness exists) Do not Downgrade | Very low (study has a small sample size <400 therefore some imprecision exists). Downgrade -1 | Undetected Do not downgrade | Outcome only found in one study, and sample size affected imprecision of data Some confounding variables accounted for but not all. | Very Low |
| Poor concentration | Felimban *et al.* 1998, n=110 participants. Rahim Anxiety Depression (RAD) questionnaire | Case-control study (non-RCT start as **low**) | Low (No serious risk of bias. Cochrane risk of bias tool is not applicable – Lack of blinding, allocation concealment, loss of follow up are not relevant)  Do not downgrade | Low (one study assessed this outcome, inconsistency is not an issue) Do not Downgrade | Low (only patients from the primary healthcare sector were included, this outcome addressed the review question, therefore no indirectness exists) Do not Downgrade | Very low (study has a small sample size <400 therefore some imprecision exists). Downgrade -1 | Undetected Do not downgrade | Outcome only found in one study, and sample size affected imprecision of data Some confounding variables accounted for but not all. | Very Low |
| Tremors | Felimban *et al.* 1998, n=110 participants. Rahim Anxiety Depression (RAD) questionnaire | Case-control study (non-RCT start as **low**) | Low (No serious risk of bias. Cochrane risk of bias tool is not applicable – Lack of blinding, allocation concealment, loss of follow up not relevant )  Do not downgrade | Low (One study assessed this outcome, inconsistency is not an issue) Do not Downgrade | Very low (only patients from the primary health care sector were included, this outcome partially addressed the review question, therefore some indirectness exists) Downgrade -1 | Very low (study has a small sample size to calculate effect size therefore some imprecision exists). Downgrade -1 | Undetected Do not downgrade | Outcome only found in one study, and sample size affected imprecision of data and some indirectess exists, Some confounding variables accounted for but not all. | Very low |
| Physical functioning | AlBuhairan *et al.* 2016, n=315 participants  Health related quality of life (HRQoL) | Cross-sectional (non-RCT start as **low**) | Low (No serious risk of bias. Cochrane risk of bias tool is not applicable – Lack of blinding, allocation concealment, loss of follow up not relevant)  Do not downgrade | Low (One study assessed this outcome, there is limited heterogeneity within the population, statistical approaches were adequate, therefore inconsistency is not an issue) Do not Downgrade | Low (This outcome addressed the review question; therefore, indirectness does not appear to be an issue) Do not Downgrade | Very Low (study has a sample size <400, therefore some imprecision exists). Downgrade -1 | Undetected Do not downgrade | Outcome only found in one study, and sample size affected imprecision of data Confounding variables were accounted for (upgrade by 1) | Low |
| Daily activities | AlBuhairan *et al.* 2016, MirRashidi *et al*. 2021, n=411 participants  Health related quality of life (HRQoL) | Cross-sectional (non-RCT start as **low**) | Low (No serious risk of bias. Cochrane risk of bias tool is not applicable – Lack of blinding, allocation concealment, loss of follow up not relevant)  Do not downgrade | Low (two studies assessed this outcome, there is some heterogeneity within the populations, statistical approaches were adequate, therefore inconsistency is not an issue) Do not Downgrade | Low (This outcome addressed the review question; therefore, indirectness does not appear to be an issue) Do not Downgrade | Low (studies have a sample size >400, therefore imprecision does not appear to be an issue). Do not downgrade | Undetected Do not downgrade | Outcome only found in two studies and Confounding variables were accounted for (upgrade by 1) | Moderate |
| Cognitive functioning | AlBuhairan *et al.* 2016, n=315 participants  Health related quality of life (HRQoL) | Cross-sectional (non-RCT start as **low**) | Low (No serious risk of bias. Cochrane risk of bias tool is not applicable – Lack of blinding, allocation concealment, loss of follow up not relevant)  Do not downgrade | Low (One study assessed this outcome, there is limited heterogeneity within the population, statistical approaches were adequate, therefore inconsistency is not an issue) Do not Downgrade | Low (This outcome addressed the review question; therefore indirectness does not appear to be an issue) Do not Downgrade | Very Low (study has a sample size <400, therefore some imprecision exists). Downgrade -1 | Undetected Do not downgrade | Outcome only found in one study, and sample size affected imprecision of data Confounding variables accounted for (upgrade by 1) | Low |
| **Emotional** | | | | | | | | | |
| Psychological  /emotional distress | Five cross-sectional studies and one case control (Felimban *et al.* 1998, AlBuhairan *et al.* 2016, Amiri *et al.* 2018, AlDubayee *et al.* 2020, Obaid *et al.* 2020, Obeidat *et al.* 2020), n=720 participants, HRQOL, SED-P, PIP, RAD, WCQ, SCL-90, PSI-SF, | Cross-sectional, case control  (non-RCT start as **low**) | Very Low (Serious risk of bias, Cochrane risk of bias tool is not applicable – Lack of blinding, allocation concealment, loss of follow up not relevant, however there are some serious limitations to the internal validity of some of the statistical methods of some studies)  Downgrade -1 | Low (Several studies assessed this outcome, there is some methodological heterogeneity in the study designs, and heterogeneity in outcome measures some inconsistency is present but is not considered as issue) Do not Downgrade | Low (This outcome addressed the review question, therefore no indirectness exists)  Do not Downgrade | Low (studies have a sample size >400 therefore imprecision does not exist). Downgrade -1 | Undetected Do not downgrade | Outcome found in several studies. Some confounding variables accounted for but not all. | Very Low |
| Parental stress | Five cross-sectional studies and 1 quasi experimental (Saghaei et al 2017, Amiri et al. 2018, AlDuabyee et al. 2020, Asadi-Shavaki et al. 2020, Obeidat et al. 2020, Khallaf et al. 2022). n=817 participants, HRQOL,RSQ PSI-SF, HPLP, PIP, PSI | Cross-sectional, case control  (non-RCT start as **low**) | Low (No serious risk of bias. Cochrane risk of bias tool is not applicable – Lack of blinding, allocation concealment, loss of follow up not relevant)  Do not downgrade | Very Low (Several studies assessed this outcome, there is methodological heterogeneity in the study designs, and heterogeneity in outcome measures. However, there little variation in the direction of the results therefore inconsistency is not considered an issue) Do not Downgrade | Low (This outcome addressed the review question, therefore no indirectness exists)  Do not Downgrade | Low (studies have a sample size >400 therefore imprecision does not exist). Downgrade -1 | Undetected Do not downgrade | Outcome found in several studies. confounding variables accounted for. (upgrade by 1) | Low |
| Emotional functioning | AlBuhairan *et al.* 2016, n=315 participants Health related quality of life (HRQoL) | Cross-sectional (non-RCT start as **low**) | Low (No serious risk of bias. Cochrane risk of bias tool is not applicable – Lack of blinding, allocation concealment, loss of follow up not relevant)  Do not downgrade | Low (One study assessed this outcome, therefore inconsistency is not an issue) Do not Downgrade | Low (This outcome addressed the review question; therefore, indirectness does not appear to be an issue) Do not Downgrade | Very Low (study has a sample size <400, therefore some imprecision exists). Downgrade -1 | Undetected Do not downgrade | Outcome only found in one study, and sample size affected imprecision of data Confounding variables accounted for (upgrade by 1) | Low |
| Depression | Felimban et al. 1998, Talakoub & Nasiri 2012, Obaid et al. 2020, n=197 participants RAD, SCL-90 | Cross-sectional, case control (non-RCT start as **low**) | Low (No serious risk of bias. Cochrane risk of bias tool is not applicable – Lack of blinding, allocation concealment, loss of follow up not relevant)  Do not downgrade | Low (three studies assessed this outcome, there is limited heterogeneity within the population, methodological heterogeneity is present and there is heterogeneity in the outcome measures used. However, there little variation in the direction of the results, therefore inconsistency is not an issue) Do not Downgrade | Low (This outcome addressed the review question; therefore, indirectness does not appear to be an issue) Do not Downgrade | Very Low (studies have a small sample size <400, therefore some imprecision exists). Downgrade -1 | Undetected Do not downgrade | Outcome only found in three studies and sample size affected imprecision of data Confounding variables not accounted for Do not upgrade | Very Low |
| Nervousness | Felimban *et al.* 1998, n=110 participants. Rahim Anxiety Depression (RAD) questionnaire | Case-control study (non-RCT start as **low**) | Low (No serious risk of bias. Cochrane risk of bias tool is not applicable – Lack of blinding, allocation concealment, loss of follow up not relevant)  Do not downgrade | Low (One study assessed this outcome, inconsistency is not an issue) Do not Downgrade | Low (only patients from the primary healthcare sector were included, this outcome addressed the review question, therefore no indirectness exists)  Do not Downgrade | Very low (study has a small sample size <400 therefore some imprecision exists). Downgrade -1 | Undetected Do not downgrade | Outcome only found in one study, and sample size affected imprecision of data Some confounding variables accounted for but not all. | Very Low |
| Irritability | Felimban *et al.* 1998, n=110 participants. Rahim Anxiety Depression (RAD) questionnaire | Case-control study (non-RCT start as **low**) | Low (No serious risk of bias. Cochrane risk of bias tool is not applicable – Lack of blinding, allocation concealment, loss of follow up not relevant)  Do not downgrade | Low (One study assessed this outcome, inconsistency is not an issue) Do not Downgrade | Low (only patients from the primary healthcare sector were included, this outcome addressed the review question, therefore no indirectness exists)  Do not Downgrade | Very low (study has a small sample size <400 therefore some imprecision exists). Downgrade -1 | Undetected Do not downgrade | Outcome only found in one study, and sample size affected imprecision of data Some confounding variables accounted for but not all. | Very Low |
| Indecision | Felimban *et al.* 1998, n=110 participants. Rahim Anxiety Depression (RAD) questionnaire | Case-control study (non-RCT start as **low**) | Low (No serious risk of bias. Cochrane risk of bias tool is not applicable – Lack of blinding, allocation concealment, loss of follow up not relevant)  Do not downgrade | Low (One study assessed this outcome, inconsistency is not an issue) Do not Downgrade | Low (only patients from the primary healthcare sector were included, this outcome addressed the review question, therefore no indirectness exists)  Do not Downgrade | Very low (study has a small sample size <400 therefore some imprecision exists). Downgrade -1 | Undetected Do not downgrade | Outcome only found in one study, and sample size affected imprecision of data Some confounding variables accounted for but not all. | Very Low |
| Primary appraisal (ability to appraise threats) Emotional-focused coping | Asadi-Shavaki et al. 2020, n=180 participants  HPLPII scale and self-developed PROM | Cross-sectional study (non-RCT start as **low**) | Low (No serious risk of bias. Cochrane risk of bias tool is not applicable – Lack of blinding, allocation concealment, loss of follow up are not relevant)  Do not downgrade | Low (one study assessed this outcome, inconsistency is not an issue) Do not Downgrade | Low (only patients from the primary healthcare sector were included, this outcome addressed the review question, therefore no indirectness exists) Do not Downgrade | Very low (study has a small sample size <400 therefore some imprecision exists). Downgrade -1 | Undetected Do not downgrade | Outcome only found in one study, and sample size affected imprecision of data Some confounding variables accounted for but not all. | Very Low |
| Secondary appraisal (ability to change stressful situations) | Asadi-Shavaki *et al*. 2020, n=180 participants  HPLPII scale and self-developed PROM | Cross-sectional study (non-RCT start as **low**) | Low (No serious risk of bias. Cochrane risk of bias tool is not applicable – Lack of blinding, allocation concealment, loss of follow up are not relevant)  Do not downgrade | Low (one study assessed this outcome, inconsistency is not an issue) Do not Downgrade | Low (This outcome addressed the review question, therefore no indirectness exists) Do not Downgrade | Very low (study has a small sample size <400 therefore some imprecision exists). Downgrade -1 | Undetected Do not downgrade | Outcome only found in one study, and sample size affected imprecision of data Some confounding variables accounted for but not all. | Very Low |
| Shock | | | | | | | | | |
| Anxiety | One cross-sectional study and one quasi-experimental (Talakoub & Nasiri 2012, Saghaei *et al.* 2017), n=173 participants, DASS, PSI-SF, SCL-90 | One cross-sectional study and one quasi-experimental  (non-RCT start as **low**) | Low (No serious risk of bias. Cochrane risk of bias tool is not applicable – Lack of blinding, allocation concealment, loss of follow up are not relevant)  Do not downgrade | Low (two studies assessed this outcome, inconsistency is not an issue) Do not Downgrade | Low (This outcome addressed the review question, therefore no indirectness exists) Do not Downgrade | Very low (the studies have a small sample size <400 therefore some imprecision exists). Downgrade -1 | Undetected Do not downgrade | Outcome only found in two studies, and sample size affected imprecision of data Some confounding variables accounted for but not all. | Very Low |
| Fear | Felimban *et al.* 1998, n=110 participants. Rahim Anxiety Depression (RAD) questionnaire | Case-control study (non-RCT start as **low**) | Low (No serious risk of bias. Cochrane risk of bias tool is not applicable – Lack of blinding, allocation concealment, loss of follow up are not relevant)  Do not downgrade | Low (one study assessed this outcome, inconsistency is not an issue) Do not Downgrade | Low (This outcome addressed the review question, therefore no indirectness exists) Do not Downgrade | Very low (study has a small sample size <400 therefore some imprecision exists). Downgrade -1 | Undetected Do not downgrade | Outcome only found in one study, and sample size affected imprecision of data Some confounding variables accounted for but not all. | Very Low |
| Hear of hypoglycaemia | Amiri *et al.* 2018 n=95 participants, HFS-P, PIP, SED-P | Cross-sectional study (non-RCT start as **low**) | Low (No serious risk of bias. Cochrane risk of bias tool is not applicable – Lack of blinding, allocation concealment, loss of follow up are not relevant)  Do not downgrade | Low (one study assessed this outcome, inconsistency is not an issue) Do not Downgrade | Low (This outcome addressed the review question, therefore no indirectness exists) Do not Downgrade | Very low (study has a small sample size <400 therefore some imprecision exists). Downgrade -1 | Undetected Do not downgrade | Outcome only found in one study, and sample size affected imprecision of data Some confounding variables accounted for but not all. | Very Low |
| Worry | AlBuhairan *et al.* 2016, n=315 participants Health related quality of life (HRQoL) | Cross-sectional (non-RCT start as **low**) | Low (No serious risk of bias. Cochrane risk of bias tool is not applicable – Lack of blinding, allocation concealment, loss of follow up not relevant)  Do not downgrade | Low (One study assessed this outcome, therefore inconsistency is not an issue) Do not Downgrade | Low (This outcome addressed the review question; therefore indirectness does not appear to be an issue) Do not Downgrade | Very Low (study has a sample size <400, therefore some imprecision exists). Downgrade -1 | Undetected Do not downgrade | Outcome only found in one study, and small sample size affected imprecision of data Confounding variables accounted for (upgrade by 1) | Low |
| Positive thinking | Khallaf *et al.* 2022, n=104 participants  RSQ | Cross-sectional (non-RCT start as **low**) | Low (No serious risk of bias. Cochrane risk of bias tool is not applicable – Lack of blinding, allocation concealment, loss of follow up not relevant)  Do not downgrade | Low (One study assessed this outcome, therefore inconsistency is not an issue) Do not Downgrade | Low (This outcome addressed the review question; therefore indirectness does not appear to be an issue) Do not Downgrade | Very Low (study has a sample size <400, therefore some imprecision exists). Downgrade -1 | Undetected Do not downgrade | Outcome only found in one study, and small sample size affected imprecision of data Confounding variables not accounted for | Very Low |
| Positive reinforcement | Mahfouz *et al.* 2018, n=92 participants  WCQ, DKQ-24 | Cross-sectional (non-RCT start as **low**) | Low (No serious risk of bias. Cochrane risk of bias tool is not applicable – Lack of blinding, allocation concealment, loss of follow up not relevant)  Do not downgrade | Low (One study assessed this outcome, therefore inconsistency is not an issue) Do not Downgrade | Low (This outcome addressed the review question; therefore indirectness does not appear to be an issue) Do not Downgrade | Very Low (study has a sample size <400, therefore some imprecision exists). Downgrade -1 | Undetected Do not downgrade | Outcome only found in one study, and small sample size affected imprecision of data. Some confounding variables not accounted for | Very Low |
| Feeling numb | Khallaf *et al.* 2022, n=104 participants  RSQ | Cross-sectional (non-RCT start as **low**) | Low (No serious risk of bias. Cochrane risk of bias tool is not applicable – Lack of blinding, allocation concealment, loss of follow up not relevant)  Do not downgrade | Low (One study assessed this outcome, therefore inconsistency is not an issue) Do not Downgrade | Low (This outcome addressed the review question; therefore indirectness does not appear to be an issue) Do not Downgrade | Very Low (study has a sample size <400, therefore some imprecision exists). Downgrade -1 | Undetected Do not downgrade | Outcome only found in one study, and small sample size affected imprecision of data Confounding variables not accounted for | Very Low |
| Lack of vitality | MirRashidi *et al.* 2021, n=96 participants  Health-related quality of life Questionnaire (SF36). | Cross-sectional (non-RCT start as **low**) | Low (No serious risk of bias. Cochrane risk of bias tool is not applicable – Lack of blinding, allocation concealment, loss of follow up not relevant)  Do not downgrade | Low (One study assessed this outcome, therefore inconsistency is not an issue) Do not Downgrade | Low (This outcome addressed the review question; therefore, indirectness does not appear to be an issue) Do not Downgrade | Very Low (study has a sample size <400, therefore some imprecision exists). Downgrade -1 | Undetected Do not downgrade | Outcome only found in one study, and small sample size affected imprecision of data. Some confounding variables not accounted for | Very Low |
| Feeling guilty | Noueiri & Nassif 2018, n=37 participants  Self-developed PROM | Cross-sectional (non-RCT start as **low**) | Low (No serious risk of bias. Cochrane risk of bias tool is not applicable – Lack of blinding, allocation concealment, loss of follow up not relevant)  Do not downgrade | Low (One study assessed this outcome, therefore inconsistency is not an issue) Do not Downgrade | Low (This outcome addressed the review question; therefore, indirectness does not appear to be an issue) Do not Downgrade | Very Low (study has a sample size <400, therefore some imprecision exists). Downgrade -1 | Undetected Do not downgrade | Outcome only found in one study, and small sample size affected imprecision of data. Some confounding variables not accounted for | Very Low |
| Avoidance | Mahfouz *et al.* 2018, n=92 participants  WCQ, DKQ-24 | Cross-sectional (non-RCT start as **low**) | Low (No serious risk of bias. Cochrane risk of bias tool is not applicable – Lack of blinding, allocation concealment, loss of follow up not relevant)  Do not downgrade | Low (One study assessed this outcome, therefore inconsistency is not an issue) Do not Downgrade | Low (This outcome addressed the review question; therefore, indirectness does not appear to be an issue) Do not Downgrade | Very Low (study has a sample size <400, therefore some imprecision exists). Downgrade -1 | Undetected Do not downgrade | Outcome only found in one study, and small sample size affected imprecision of data. Some confounding variables not accounted for | Very Low |
| Confrontation | Mahfouz *et al.* 2018, n=92 participants  WCQ, DKQ-24 | Cross-sectional (non-RCT start as **low**) | Low (No serious risk of bias. Cochrane risk of bias tool is not applicable – Lack of blinding, allocation concealment, loss of follow up not relevant)  Do not downgrade | Low (One study assessed this outcome, therefore inconsistency is not an issue) Do not Downgrade | Low (This outcome addressed the review question; therefore, indirectness does not appear to be an issue) Do not Downgrade | Very Low (study has a sample size <400, therefore some imprecision exists). Downgrade -1 | Undetected Do not downgrade | Outcome only found in one study, and small sample size affected imprecision of data. Some confounding variables not accounted for | Very Low |
| Burden of care | Obaid *et al.* 2020, n=52 participants, self-developed PROM. | Cross-sectional (non-RCT start as **low**) | Very Low (Serious risk of bias, Cochrane risk of bias tool is not applicable – Lack of blinding, allocation concealment, loss of follow up not relevant, however there are some serious limitations to the internal validity of the statistical methods of this study)  Downgrade -1 | Low (One study assessed this outcome, therefore inconsistency is not an issue) Do not Downgrade | Low (This outcome addressed the review question; therefore, indirectness does not appear to be an issue) Do not Downgrade | Very Low (study has a sample size <400, therefore some imprecision exists). Downgrade -1 | Undetected Do not downgrade | Outcome only found in one study, and small sample size affected imprecision of data. Some confounding variables not accounted for | Very Low |
| Hope | | | | | | | | | |
| Coping | Three cross-sectional studies and one quasi-experimental (Saghaei *et al.* 2017, Mahfouz *et al.* 2018, Asadi-Shavaki *et al.* 2020, Khallaf *et al.* 2022), n=460 participants, WCQ, HPLPII, PSI-SF, SED-P | Cross-sectional and quasi-experimental (non-RCT start as **low**) | Low (No serious risk of bias. Cochrane risk of bias tool is not applicable – Lack of blinding, allocation concealment, loss of follow up not relevant)  Do not downgrade | Low (Four studies assessed this outcome, there is methodological heterogeneity between study designs, and outcome measures used but inconsistency is not an issue) Do not Downgrade | Low (This outcome addressed the review question; therefore, indirectness does not appear to be an issue) Do not Downgrade | Low (studies have a sample size >400, therefore imprecision does not appear to be an issue). Do not Downgrade | Undetected Do not downgrade | Outcome found in four studies, and sample size affected imprecision of data. Some confounding variables not accounted for | Very Low |
| Self-efficacy | Amiri *et al.* 2018, n=61 participants  HFS-P, PIP, SED-P | Cross-sectional (non-RCT start as **low**) | Low (No serious risk of bias. Cochrane risk of bias tool is not applicable – Lack of blinding, allocation concealment, loss of follow up not relevant)  Do not downgrade | Low (One study assessed this outcome, therefore inconsistency is not an issue) Do not Downgrade | Low (This outcome addressed the review question; therefore, indirectness does not appear to be an issue) Do not Downgrade | Very Low (study has a sample size <400, therefore some imprecision exists). Downgrade -1 | Undetected Do not downgrade | Outcome only found in one study, and small sample size affected imprecision of data. All confounding variables accounted for  Upgrade +1 | Low |
| Self-empowerment | | | | | | | | | |
| Self-control | Mahfouz *et al.* 2018, n=92 participants  WCQ, DKQ-24 | Cross-sectional (non-RCT start as **low**) | Low (No serious risk of bias. Cochrane risk of bias tool is not applicable – Lack of blinding, allocation concealment, loss of follow up not relevant)  Do not downgrade | Low (One study assessed this outcome, therefore inconsistency is not an issue) Do not Downgrade | Low (This outcome addressed the review question; therefore, indirectness does not appear to be an issue) Do not Downgrade | Very Low (study has a sample size <400, therefore some imprecision exists). Downgrade -1 | Undetected Do not downgrade | Outcome only found in one study, and small sample size affected imprecision of data. Some confounding variables not accounted for | Very Low |
| Quality of life | Three cross-sectional studies and one case control (AlBuhairan et al. 2016, Noueiri & Nassif 2018, Hashemipour-Zavareh et al. 2020, MirRashidi et al. 2021), n=548 participants, HRQoL, FQoL and self-developed PROM | Cross-sectional and case control (non-RCT start as **low**) | Low (No serious risk of bias. Cochrane risk of bias tool is not applicable – Lack of blinding, allocation concealment, loss of follow up not relevant)  Do not downgrade | Low (Four studies assessed this outcome, there is methodological heterogeneity between study designs, and outcome measures used but inconsistency is not an issue) Do not Downgrade | Low (This outcome addressed the review question; therefore, indirectness does not appear to be an issue) Do not Downgrade | Low (studies have a sample size >400, therefore imprecision does not appear to be an issue). Do not Downgrade | Undetected Do not downgrade | Outcome found in four studies, and sample size affected imprecision of data. Some confounding variables not accounted for not all | Low |
| Knowledge and diabetes control | Khallaf *et al.* 2022, n=104 participants  RSQ | Cross-sectional (non-RCT start as **low**) | Low (No serious risk of bias. Cochrane risk of bias tool is not applicable – Lack of blinding, allocation concealment, loss of follow up not relevant)  Do not downgrade | Low (One study assessed this outcome, therefore inconsistency is not an issue) Do not Downgrade | Low (This outcome addressed the review question; therefore, indirectness does not appear to be an issue) Do not Downgrade | Very Low (study has a sample size <400, therefore some imprecision exists). Downgrade -1 | Undetected Do not downgrade | Outcome only found in one study, and small sample size affected imprecision of data. Some Confounding variables accounted for not all | Very Low |
| Knowledge and coping |  |  |  |  |  |  |  |  |  |
| **Social** | | | | | | | | | |
| Financial challenges | Three cross-sectional studies and one case control (Mahfouz et al. 2018, Noueiri & Nassif 2018, Hashemipour-Zavareh *et al.* 2020, MirRashidi *et al.* 2021), n=325 participants, FQoL, WCQ, DKQ-24,self-developed PROM | Cross-sectional and case control (non-RCT start as **low**) | Low (No serious risk of bias. Cochrane risk of bias tool is not applicable – Lack of blinding, allocation concealment, loss of follow up not relevant)  Do not downgrade | Low (Four studies assessed this outcome, there is methodological heterogeneity between study designs, and outcome measures used but inconsistency is not an issue) Do not Downgrade | Low (This outcome addressed the review question; therefore, indirectness does not appear to be an issue) Do not Downgrade | Very Low (studies have a small sample size <400, therefore imprecision exists). Downgrade-1 | Undetected Do not downgrade | Outcome found in four studies, and sample size affected imprecision of data. Some confounding variables not accounted for not all | Very Low |
| Sense of responsibility | | | | | | | | | |
| Communication | AlBuhairan *et al.* 2016, AlDubayee *et al.* 2020, n=635 participants  HRQoL, PIP | Cross-sectional (non-RCT start as **low**) | Low (No serious risk of bias. Cochrane risk of bias tool is not applicable – Lack of blinding, allocation concealment, loss of follow up not relevant)  Do not downgrade | Low (Two studies assessed this outcome, there is heterogeneity between the outcome measures used but inconsistency is not an issue) Do not Downgrade | Low (This outcome addressed the review question; therefore, indirectness does not appear to be an issue) Do not Downgrade | Low (studies have an adequate sample size >400, therefore imprecision is not an issue). Do not downgrade | Undetected Do not downgrade | Outcome found in two studies, and sample size affected imprecision of data. All confounding variables accounted for  (upgrade +1) | Low |
| Family equilibrium | | | | | | | | | |
| Role/ family function | Amiri *et al.* 2018, AlDubayee *et al.* 2020, n= 381 participants  PIP, | Cross-sectional (non-RCT start as **low**) | Low (No serious risk of bias. Cochrane risk of bias tool is not applicable – Lack of blinding, allocation concealment, loss of follow up not relevant)  Do not downgrade | Low (Two studies assessed this outcome, there is homogeneity between the outcome measures used therefore inconsistency is not an issue) Do not Downgrade | Low (This outcome addressed the review question; therefore, indirectness does not appear to be an issue) Do not Downgrade | Very Low (studies have a small sample size <400, therefore imprecision is an issue). Do not downgrade | Undetected Do not downgrade | Outcome found in two studies, and sample size affected imprecision of data. All confounding variables accounted for  (upgrade +1) | Low |
| Support from family | Mahfouz *et al.* 2018, n=92 participants  WCQ, DKQ-24 | Cross-sectional (non-RCT start as **low**) | Low (No serious risk of bias. Cochrane risk of bias tool is not applicable – Lack of blinding, allocation concealment, loss of follow up not relevant)  Do not downgrade | Low (One study assessed this outcome, therefore inconsistency is not an issue) Do not Downgrade | Low (This outcome addressed the review question; therefore, indirectness does not appear to be an issue) Do not Downgrade | Very Low (study has a sample size <400, therefore some imprecision exists). Downgrade -1 | Undetected Do not downgrade | Outcome only found in one study, and small sample size affected imprecision of data. Some confounding variables not accounted for | Very Low |
| Support from school | | | | | | | | | |
| Support from HCPs | Mahfouz *et al.* 2018, n=92 participants  WCQ, DKQ-24 | Cross-sectional (non-RCT start as **low**) | Low (No serious risk of bias. Cochrane risk of bias tool is not applicable – Lack of blinding, allocation concealment, loss of follow up not relevant)  Do not downgrade | Low (One study assessed this outcome, therefore inconsistency is not an issue) Do not Downgrade | Low (This outcome addressed the review question; therefore, indirectness does not appear to be an issue) Do not Downgrade | Very Low (study has a sample size <400, therefore some imprecision exists). Downgrade -1 | Undetected Do not downgrade | Outcome only found in one study, and small sample size affected imprecision of data. Some confounding variables not accounted for | Very Low |
| Mistrust of HCPs and diabetes treatment | | | | | | | | | |
| Quality of diabetes related medical care | Amiri et al. 2018, AlDubayee et al. 2020, n= 431 participants  PIP,SED-P | Cross-sectional (non-RCT start as **low**) | Low (No serious risk of bias. Cochrane risk of bias tool is not applicable – Lack of blinding, allocation concealment, loss of follow up not relevant)  Do not downgrade | Low (Two studies assessed this outcome, therefore inconsistency is not an issue) Do not Downgrade | Low (This outcome addressed the review question; therefore indirectness does not appear to be an issue) Do not Downgrade | Low (studies have an adequate sample size >400, therefore imprecision is not an issue). Do not Downgrade | Undetected Do not downgrade | Outcome found in two studies, and sample size was Some confounding variables were not accounted for | Low |
| Support and respect from society | Hashemipour-Zavareh et al. 2020, n=100 participants | Case- control (non-RCT start as **low**) | Low (No serious risk of bias. Cochrane risk of bias tool is not applicable – Lack of blinding, allocation concealment, loss of follow up not relevant)  Do not downgrade | Low (One study assessed this outcome, therefore inconsistency is not an issue) Do not Downgrade | Low (This outcome addressed the review question; therefore, indirectness does not appear to be an issue) Do not Downgrade | Very Low (study has a sample size <400, therefore some imprecision exists). Downgrade -1 | Undetected Do not downgrade | Outcome only found in one study, and small sample size affected imprecision of data. Some confounding variables not accounted for | Very Low |
| Social functioning | AlBuhairan *et al.* 2016, n=315 participants  Health related quality of life (HRQoL) | Cross-sectional (non-RCT start as **low**) | Low (No serious risk of bias. Cochrane risk of bias tool is not applicable – Lack of blinding, allocation concealment, loss of follow up not relevant)  Do not downgrade | Low (One study assessed this outcome, there is limited heterogeneity within the population, statistical approaches were adequate, therefore inconsistency is not an issue) Do not Downgrade | Low (This outcome addressed the review question; therefore, indirectness does not appear to be an issue) Do not Downgrade | Very Low (study has a sample size <400, therefore some imprecision exists). Downgrade -1 | Undetected Do not downgrade | Outcome only found in one study, and sample size affected imprecision of data Confounding variables accounted for (upgrade by 1) | Low |
| Family functioning | AlBuhairan *et al.* 2016, n=315 participants  Health related quality of life (HRQoL) | Cross-sectional (non-RCT start as **low**) | Low (No serious risk of bias. Cochrane risk of bias tool is not applicable – Lack of blinding, allocation concealment, loss of follow up not relevant)  Do not downgrade | Low (One study assessed this outcome, there is limited heterogeneity within the population, statistical approaches were adequate, therefore inconsistency is not an issue) Do not Downgrade | Low (This outcome addressed the review question; therefore, indirectness does not appear to be an issue) Do not Downgrade | Very Low (study has a sample size <400, therefore some imprecision exists). Downgrade -1 | Undetected Do not downgrade | Outcome only found in one study, and sample size affected imprecision of data Confounding variables accounted for (upgrade by 1) | Low |
| Family relationships | AlBuhairan *et al.* 2016, n=315 participants  Health related quality of life (HRQoL) | Cross-sectional (non-RCT start as **low**) | Low (No serious risk of bias. Cochrane risk of bias tool is not applicable – Lack of blinding, allocation concealment, loss of follow up not relevant)  Do not downgrade | Low (One study assessed this outcome, there is limited heterogeneity within the population, statistical approaches were adequate, therefore inconsistency is not an issue) Do not Downgrade | Low (This outcome addressed the review question; therefore, indirectness does not appear to be an issue) Do not Downgrade | Very Low (study has a sample size <400, therefore some imprecision exists). Downgrade -1 | Undetected Do not downgrade | Outcome only found in one study, and sample size affected imprecision of data Confounding variables accounted for (upgrade by 1) | Low |
| Social isolation | | | | | | | | | |
| Impact on social life | Hashemipour-Zavareh *et al.* 2020, MirRashidi *et al.* 2021, n=196 participants | Case-control, Cross-sectional (non-RCT start as **low**) | Low (No serious risk of bias. Cochrane risk of bias tool is not applicable – Lack of blinding, allocation concealment, loss of follow up not relevant)  Do not downgrade | Low (Two studies assessed this outcome, therefore inconsistency is not an issue) Do not Downgrade | Low (This outcome addressed the review question; therefore, indirectness does not appear to be an issue) Do not Downgrade | Very Low (studies have a small sample size <400, therefore some imprecision exists). Downgrade -1 | Undetected Do not downgrade | Outcome found in two studies, and sample size was Some confounding variables were not accounted for | Very Low |
| Social burden | Obaid et al 2020, n=52 participants | Cross-sectional (non-RCT start as **low**) | Low (No serious risk of bias. Cochrane risk of bias tool is not applicable – Lack of blinding, allocation concealment, loss of follow up not relevant)  Do not downgrade | Very Low (One study assessed this outcome, statistical approaches were inadequate, therefore inconsistency is an issue)  Downgrade -1 | Low (This outcome addressed the review question; therefore, indirectness does not appear to be an issue) Do not Downgrade | Very Low (study has a sample size <400, therefore some imprecision exists). Downgrade -1 | Undetected Do not downgrade | Outcome only found in one study, and sample size affected imprecision of data Confounding accounted for  (upgrade +1) | Very Low |
| Social stigma | | | | | | | | | |
| Social discrimination | | | | | | | | | |
| Separation anxiety | Noueiri & Nassif 2018, n=37 participants  Self-developed PROM | Cross-sectional (non-RCT start as **low**) | Low (No serious risk of bias. Cochrane risk of bias tool is not applicable – Lack of blinding, allocation concealment, loss of follow up not relevant)  Do not downgrade | Low (One study assessed this outcome, there is limited heterogeneity within the population, statistical approaches were adequate, therefore inconsistency is not an issue) Do not Downgrade | Low (This outcome addressed the review question; therefore, indirectness does not appear to be an issue) Do not Downgrade | Very Low (study has a sample size <400, therefore some imprecision exists). Downgrade -1 | Undetected Do not downgrade | Outcome only found in one study, and sample size affected imprecision of data Confounding variables not accounted for | Very Low |
| **Spiritual** | | | | | | | | | |
| Spiritual role in accepting diagnosis | Khallaf *et al.* 2022, n=104 participants  RSQ | Cross-sectional (non-RCT start as **low**) | Low (No serious risk of bias. Cochrane risk of bias tool is not applicable – Lack of blinding, allocation concealment, loss of follow up not relevant)  Do not downgrade | Low (One study assessed this outcome, therefore inconsistency is not an issue) Do not Downgrade | Low (This outcome addressed the review question; therefore, indirectness does not appear to be an issue) Do not Downgrade | Very Low (study has a sample size <400, therefore some imprecision exists). Downgrade -1 | Undetected Do not downgrade | Outcome only found in one study, and small sample size affected imprecision of data Confounding variables not accounted for | Very Low |
| Spiritual role in coping | | | | | | | | | |
| God’s will/destiny | | | | | | | | | |
| Spiritual support | | | | | | | | | |

Supplementary Table 10 GRADE-CERQual assessment

| **GRADE-CERQual** | | | | | | | | | | |
| --- | --- | --- | --- | --- | --- | --- | --- | --- | --- | --- |
| **Population:** Parents of children with T1DM | | | | | | | | | | |
| **Setting:** MENA region | | | | | | | | | | |
|  | **Study Contributing to outcome** | **Study design** | **Methodological limitations** | **Coherence** | **Adequacy of data** | | **Relevance** | **Quality of data** | **Comments** | |
| **Outcomes** | | | | | | | | | | |
| **Physical** | | | | | | | | | | |
| Insomnia/sleep | Asaad *et al.* 2022, reported this finding, n=11 participants | Phenomenology | No or minor concerns about methodological limitations (study addressed the reflexivity of the researcher as to their influence on the participants and vice-versa ) | Moderate concerns (There was a clear fit between the data from the primary study and the review finding, and the finding reflects the complexity and variation of the data. However, patterns across studies cannot be determined from one study | Serious concerns (This finding was only found in one study moderate concerns regarding the adequacy of the data leading low confidence in the use of this finding across all studies | | No concerns (the outcome is relevant to the study) (population, context, setting) | Very Low | This finding was only found in one study where there were minor concerns regarding methodological limitations and moderate concerns regarding the coherence and serious concerns regarding the adequacy of the data leading to very low confidence in the use of this finding across all studies | |
| Fatigue | Povlsen & Ringsberg 2008, reported this finding, n=7 participants | Phenomenology | Moderate concerns about methodological limitations (Study failed to address the reflexivity of the researcher as to their influence on the participants and vice-versa was not clear)no mention of data saturation | Moderate concerns (the context of the study was relevant to the review finding, but only one study addressed it. | Serious concerns (the finding is supported by data from only one study, and there were relatively thin data (no quotations) to support it) | | No concerns (the context of the study was relevant to the review finding. (population, context, setting) | Very Low | This finding was only found in one study where there were moderate concerns regarding the methods and the coherence and serious concerns regarding the adequacy of the data leading to very low confidence in the use of this finding across all studies | |
| Poor concentration | | | | | | | | | | |
| Tremors | | | | | | | | | | |
| Physical functioning | | | | | | | | | | |
| Daily activities | | | | | | | | | | |
| Cognitive functioning | | | | | | | | | | |
| **Emotional** | | | | | | | | | | |
| Psychological  /emotional distress | Seven studies (Povlsen & Ringsberg 2008, Oskouie *et al.* 2013, Khandan *et al.* 2018 (a), Asaad *et al.* 2022, Moghadam *et al.* 2022, Momani *et al*. 2022) reported this finding. n=85 participants. | Grounded theory, descriptive phenomenological research, Yin's qualitative data analysis | Minor concerns (Some studies does not have a clear philosophical/ theoretical perspective, most of the studies failed to address reflexivity of the researcher as to their influence on the participants and vice-versa was not clear and data collection and recruitment methods) | No concerns about coherence | No or very minor concerns about adequacy and the finding was found across seven studies | | No concerns about relevance the finding is relative to the review question. (population, context, setting) | Moderate | This finding was found across seven studies with some minor methodological limitations and was found to be coherent, relevant and had adequacy of data leading to moderate confidence in the use of this finding across all studies. | |
| Parental stress | Khandan *et al.* 2018 (a), Moghadam *et al.* 2022, reported this finding, n=31 participants | Content analysis | Minor concerns about methodological limitations (related to the reflexivity of the researcher as to their influence on the participants and vice-versa was not clear) | No or very minor concerns about coherence | Moderate concerns about adequacy of data the finding was only found in two studies | | No or very minor concerns about relevance (population, context, setting) | Low | This finding was found across two studies with minor concerns regarding methodological limitations and adequacy of data because of the number of studies and the thin data. However, there were no concerns regarding coherence and relevance. Leading to low confidence in the use of this finding across all studies. | |
| Emotional functioning | | | | | | | | | | |
| Depression | | | | | | | | | | |
| Nervousness | Khandan *et al.* 2018 (b) reported this finding, n=15 participants | Content analysis | Minor concerns about methodological limitations (related to the reflexivity of the researcher as to their influence on the participants and vice-versa was not clear and methods of recruitment) | Moderate concerns about coherence as finding was found in one study with thin data | Serious concerns about adequacy of data the finding was only found in one study | | No or very minor concerns about relevance (population, context, setting) | Very Low | This finding was found in one study with some minor methodological limitations and some moderate concerns about coherence however there were serious concerns regarding adequacy of data and no concerns regarding relevance Therefore there is very low confidence in the use of this finding across all studies | |
| Irritability | | | | | | | | | | |
| Indecision | | | | | | | | | | |
| Primary appraisal (ability to appraise threats) Emotional-focused coping | | | | | | | | | | |
| Secondary appraisal (ability to change stressful situations) | | | | | | | | | | |
| Shock | Povlsen & Ringsberg 2008, Khandan *et al.* 2018 (b), Rossiter *et al.* 2019, Momani *et al.* 2022, Asaad *et al.* 2022, reported this finding, n=56 participants | Grounded theory, content analysis, phenomenology, Yin's qualitative data analysis | Minor concerns about methodological limitations (related to the reflexivity of the researcher as to their influence on the participants and vice-versa was not clear and methods of recruitment) | No or minor concerns (There was a clear fit between the data from the primary studies and the review finding, and the finding reflects the complexity and variation of the data.) | No or minor concerns as the finding was found across six studies | | No or minor concerns the finding was relevant to the review question (population, context, setting) | Moderate | This finding was found across six studies with some minor methodological limitations and no concerns about coherence or adequacy of data or relevance Therefore there is moderates confidence in the use of this finding across all studies | |
| Disbelief | Rossiter *et al.* 2019, reported this finding, n=4 participants | Yin's qualitative data analysis | Minor concerns about methodological limitations (related to the reflexivity of the researcher as to their influence on the participants and vice-versa was not clear and methods of recruitment) | Moderate concerns about coherence as finding was found in one study with thin data | Serious concerns about adequacy of data the finding was only found in one study | | No or minor concerns the finding was relevant to the review question (population, context, setting) | Very Low | This finding was found in one study with some minor methodological limitations and some moderate concerns about coherence however there were serious concerns regarding adequacy of data and no concerns regarding relevance Therefore there is very low confidence in the use of this finding across all studies | |
| Anxiety | Povlsen & Ringsberg 2008, reported this finding, n=7 participants | Phenomenology | Minor concerns about methodological limitations (related to the reflexivity of the researcher as to their influence on the participants and vice-versa was not clear and methods of recruitment) | Moderate concerns about coherence as finding was found in one study with thin data to support it | Serious concerns about adequacy of data the finding was only found in one study | | No or minor concerns the finding was relevant to the review question (population, context, setting) | Very Low | This finding was found in one study with some minor methodological limitations and some moderate concerns about coherence however there were serious concerns regarding adequacy of data and no concerns regarding relevance Therefore there is very low confidence in the use of this finding across all studies | |
| Fear | Khandan *et al.* 2018 (a), reported this finding, n=11 | Descriptive phenomenological research | Minor concerns about methodological limitations (related to the reflexivity of the researcher as to their influence on the participants and vice-versa was not clear and methods of recruitment) | Moderate concerns about coherence as finding was found in one study | Serious concerns about adequacy of data the finding was only found in one study. | | No or minor concerns the finding was relevant to the review question (population, context, setting) | Very Low | This finding was found in one study with some minor methodological limitations and some moderate concerns about coherence however there were serious concerns regarding adequacy of data and no concerns regarding relevance Therefore there is very low confidence in the use of this finding across all studies | |
| Fear of hypoglycaemia | Asaad *et al.* 2022 reported this finding, n=11 | Phenomenology | Minor concerns about methodological limitations (related to the reflexivity of the researcher as to their influence on the participants and vice-versa was not clear and methods of recruitment) | Moderate concerns about coherence as finding was found in one study with thin data to support it. | Serious concerns about adequacy of data the finding was only found in one study | | No or minor concerns the finding was relevant to the review question (population, context, setting) | Very Low | This finding was found in one study with some minor methodological limitations and some moderate concerns about coherence however there were serious concerns regarding adequacy of data and no concerns regarding relevance Therefore there is very low confidence in the use of this finding across all studies | |
| Worry (future, marriage) | Seven studies (Povlsen & Ringsberg 2008, Oskouie *et al.* 2013, Elissa *et al*. 2017, Khandan *et al.* 2018 (a, b), Rossiter *et al.* 2019, Momani *et al.* 2022) reported this finding. n=83 participants. | Grounded theory, descriptive phenomenological research, content analysis, Yin's qualitative data analysis | Minor concerns about methodological limitations (related to the reflexivity of the researcher as to their influence on the participants and vice-versa was not clear and methods of recruitment) | No or minor concerns (There was a clear fit between the data from the primary studies and the review finding, and the finding reflects the complexity and variation of the data.) | No or minor concerns as the finding was found across seven studies | | No or minor concerns the finding was relevant to the review question (population, context, setting) | Moderate | This finding was found across seven studies with some minor methodological limitations and no concerns about coherence or adequacy of data or relevance Therefore there is moderates confidence in the use of this finding across all studies | |
| Positive thinking | | | | | | | | | | |
| Positive reinforcement | | | | | | | | | | |
| Feeling numb | | | | | | | | | | |
| Lack of vitality | | | | | | | | | | |
| Feeling guilty | Povlsen & Ringsberg 2008, Khandan *et al.* 2018 (b), reported this finding, n=22 participants | Phenomenology, content analysis | Minor concerns about methodological limitations (related to the reflexivity of the researcher as to their influence on the participants and vice-versa was not clear and methods of recruitment | Moderate concerns about coherence as finding was found in two studies with thin data | Serious concerns about adequacy of data the finding was only found in two studies. | | No or minor concerns the finding was relevant to the review question (population, context, setting) | Very Low | This finding was found in two studies with some minor methodological limitations and some moderate concerns about coherence however there were serious concerns regarding adequacy of data and no concerns regarding relevance Therefore there is very low confidence in the use of this finding across all studies | |
| Avoidance | | | | | | | | | | |
| Confrontation | | | | | | | | | | |
| Hope | Povlsen & Ringsberg 2008, reported this finding, n=7 participants | Phenomenology | Minor concerns about methodological limitations (related to the reflexivity of the researcher as to their influence on the participants and vice-versa was not clear and methods of recruitment | Moderate concerns about coherence as finding was found in one study with thin data | Serious concerns about adequacy of data the finding was only found in one study | | No or minor concerns the finding was relevant to the review question (population, context, setting) | Very Low | This finding was found in one study with some minor methodological limitations and some moderate concerns about coherence however there were serious concerns regarding adequacy of data and no concerns regarding relevance Therefore there is very low confidence in the use of this finding across all studies | |
| Burden of care | Two studies (Khandan *et al.* 2018 (a), Moghadam *et al.* 2022) reported this finding. n=31 participants. | Descriptive phenomenological research, content analysis | Minor concerns about methodological limitations (related to the reflexivity of the researcher as to their influence on the participants and vice-versa was not clear and methods of recruitment | Minor concerns about coherence as finding was found in two studies with thin data | Serious concerns about adequacy of data the finding was only found in two studies, with thin data to support it. | | No or minor concerns the finding was relevant to the review question (population, context, setting) | Very Low | This finding was found in two studies with some minor methodological limitations and some moderate concerns about coherence however there were serious concerns regarding adequacy of data and no concerns regarding relevance Therefore there is very low confidence in the use of this finding across all studies | |
| Coping | Oskouie *et al.* 2013, Asaad *et al.* 2022, Moghadam *et al.* 2022, reported this finding, n=48 participants | Grounded theory, content analysis, phenomenology | Minor concerns about methodological limitations (related to the reflexivity of the researcher as to their influence on the participants and vice-versa was not clear and methods of recruitment | Very minor concerns (There was a clear fit between the data from the primary studies and the review finding, and sufficiently reflected the complexity and variation of the data) | Minor concerns about adequacy of data, finding was found in three studies with rich data | | No or minor concerns the finding was relevant to the review question (population, context, setting) | Moderate | This finding was found in three studies with some minor methodological limitations and some minor concerns about coherence and adequacy of data and no concerns regarding relevance Therefore there is moderate confidence in the use of this finding across all studies | |
| Self-efficacy | | | | | | | | | | |
| Self-empowerment | Moghadam *et al.* 2022, reported this finding, n=20 participants | Content analysis | Minor concerns about methodological limitations (related to the reflexivity of the researcher as to their influence on the participants and vice-versa was not clear and methods of recruitment | Moderate concerns (There was a clear fit between the data from the primary study and the review finding, however the fining was only found in one study impacting the reflection of the complexity and variation of the data.) | Serious concerns about adequacy of data as it was found in one study | | No or minor concerns the finding was relevant to the review question (population, context, setting) | Very Low | This finding was found in one study with some minor methodological limitations and some moderate concerns about coherence however there were serious concerns regarding adequacy of data and no concerns regarding relevance Therefore there is very low confidence in the use of this finding across all studies | |
| Self-control | | | | | | | | | | |
| Quality of life | | | | | | | | | | |
| Knowledge and diabetes control | Khandan *et al.* 2018 (a, b), reported this finding, n=26 participants | Descriptive phenomenological research, content analysis | Minor concerns about methodological limitations (related to the reflexivity of the researcher as to their influence on the participants and vice-versa was not clear and methods of recruitment | Moderate concerns (There was a clear fit between the data from the primary studies and the review finding, and the finding reflects the complexity and variation of the data, however only two studies contributed to this finding) | Moderate concerns (The finding is supported by data from two studies and there are sufficient data to support it.) | | No or minor concerns the finding was relevant to the review question (population, context, setting) | Very Low | This finding was found in two studies with some minor methodological limitations and some moderate concerns about coherence and moderate concerns regarding adequacy of data and no concerns regarding relevance Therefore there is very low confidence in the use of this finding across all studies | |
| Knowledge and coping | Khandan *et al.* 2018 (a), Asaad *et al.* 2022, Moghadam *et al.* 2022, reported this finding, n=47 participants | Content analysis, phenomenology | Minor concerns about methodological limitations (related to the reflexivity of the researcher as to their influence on the participants and vice-versa was not clear and methods of recruitment | Very minor concerns (There was a clear fit between the data from the primary studies and the review finding, and the finding reflects the complexity and variation of the data.) | Minor concerns (The finding is supported by data from three studies and there are sufficient data to support it.) | | No or minor concerns the finding was relevant to the review question (population, context, setting) | Moderate | This finding was found in three studies with some minor methodological limitations and minor concerns about coherence and adequacy of data and no concerns regarding relevance Therefore there is moderate confidence in the use of this finding across all studies | |
| **Social** | | | | | | | | | | |
| Financial challenges | Eight studies (Povlsen & Ringsburg 2008, Oskouie *et al.* 2013, Elissa *et al.* 2017, Khandan *et al.* 2018 (a, b), Rossiter et al. 2019, Asaad et al. 2022, Moghadam *et al.* 2022) reported this findings. n=95 participants. | Grounded theory, descriptive phenomenological research, content analysis, Yin's qualitative data analysis | Minor concerns about methodological limitations (related to the reflexivity of the researcher as to their influence on the participants and vice-versa was not clear and methods of recruitment | Very minor concerns (There was a clear fit between the data from the primary studies and the review finding, and the finding reflects the complexity and variation of the data.) | Minor concerns (The finding is supported by data from eight studies and there are sufficient data to support it.) | | No or minor concerns the finding was relevant to the review question (population, context, setting) | Moderate | This finding was found in eight studies with some minor methodological limitations and minor concerns about coherence and adequacy of data and no concerns regarding relevance Therefore there is moderate confidence in the use of this finding across all studies | |
| Sense of responsibility | | | | | | | | | | |
| Communication | | | | | | | | | | |
| Family equilibrium | Moghadam *et al.* 2022, reported this finding, n=20 participants | Content analysis | Minor concerns about methodological limitations (related to the reflexivity of the researcher as to their influence on the participants and vice-versa was not clear and methods of recruitment | Moderate concerns (There was a clear fit between the data from the primary studies and the review finding, and the finding reflects the complexity and variation of the data, however only one study contributed to this finding) | Serious concerns (The finding is supported by data from one study and there are sufficient data to support it.) | | No or minor concerns the finding was relevant to the review question (population, context, setting) | Very Low | This finding was found in one study with some minor methodological limitations and some moderate concerns about coherence and serious concerns regarding adequacy of data and no concerns regarding relevance Therefore there is very low confidence in the use of this finding across all studies | |
| Role/ family function | | | | | | | | | | |
| Support from family | Khandan *et al.* 2018 (b), Rossiter *et al.* 2019, Asaad *et al.* 2022, Moghadam *et al.* 2022, Momani *et al.* 2022, reported this finding, n=69 participants | Descriptive phenomenological research, grounded theory, content analysis, Yin's qualitative data analysis | Minor concerns about methodological limitations (related to the reflexivity of the researcher as to their influence on the participants and vice-versa was not clear and methods of recruitment | Very minor concerns (There was a clear fit between the data from the primary studies and the review finding, and the finding reflects the complexity and variation of the data.) | Minor concerns (The finding is supported by data from five studies and there are sufficient data to support it.) | | No or minor concerns the finding was relevant to the review question (population, context, setting) | Moderate | This finding was found in five studies with some minor methodological limitations and minor concerns about coherence and adequacy of data and no concerns regarding relevance Therefore there is moderate confidence in the use of this finding across all studies | |
| Support from school | Rossiter *et al.* 2019, Asaad *et al.* 2022, reported this finding, n=15 participants | Asaad et al. 2022, Rossiter et al. 2019 reported this finding, n=15 participants | Minor concerns about methodological limitations (related to the reflexivity of the researcher as to their influence on the participants and vice-versa was not clear and methods of recruitment | Minor concerns (There was a clear fit between the data from the primary studies and the review finding, and the finding reflects the complexity and variation of the data, however only two studies contributed to this finding) | Serious concerns (The finding is supported by data from two studies and there are sufficient data to support it.) | | No or minor concerns the finding was relevant to the review question (population, context, setting) | Low | This finding was found in two studies with some minor methodological limitations and some minor concerns about coherence and serious concerns regarding adequacy of data and no concerns regarding relevance Therefore there is low confidence in the use of this finding across all studies | |
| Support from HCPs | Oskouie *et al.* 2013, Khandan *et al.* 2018 (b), Rossiter *et al.* 2019, Asaad *et al.* 2022, Moghadam *et al.* 2022, reported this finding, n=67 participants | Grounded theory, descriptive phenomenological research, content analysis, Yin's qualitative data analysis | Minor concerns about methodological limitations (related to the reflexivity of the researcher as to their influence on the participants and vice-versa was not clear and methods of recruitment | Very minor concerns (There was a clear fit between the data from the primary studies and the review finding, and the finding reflects the complexity and variation of the data.) | Minor concerns (The finding is supported by data from five studies and there are sufficient data to support it.) | | No or minor concerns the finding was relevant to the review question (population, context, setting) | Moderate | This finding was found in five studies with some minor methodological limitations and minor concerns about coherence and adequacy of data and no concerns regarding relevance Therefore there is moderate confidence in the use of this finding across all studies | |
| Mistrust of HCPs and diabetes treatment | | | | | | | | | | |
| Quality of diabetes related medical care | | | | | | | | | | |
| Support and respect from society | Khandan *et al.* 2018 (b), Moghadam *et al.* 2022, Momani *et al.* 2022, reported this finding, n=54 participants | Grounded theory, content analysis | Minor concerns about methodological limitations (related to the reflexivity of the researcher as to their influence on the participants and vice-versa was not clear and methods of recruitment | No or minor concerns (There was a clear fit between the data from the primary study and the review finding, and the finding reflects the complexity and variation of the data.) | Minor concerns (The finding is supported by data from three studies, and some have thin data to support it | | No or minor concerns the finding was relevant to the review question (population, context, setting) | Moderate | This finding was found in three studies with some minor methodological limitations and minor concerns about coherence and adequacy of data and no concerns regarding relevance Therefore there is moderate confidence in the use of this finding across all studies | |
| Social functioning | | | | | | | | | | |
| Family functioning | | | | | | | | | | |
| Family relationships | Moghadam *et al.* 2022, reported this finding, n= 20 participants | Content analysis | Minor concerns about methodological limitations (related to the reflexivity of the researcher as to their influence on the participants and vice-versa was not clear and methods of recruitment | Moderate concerns (There was a clear fit between the data from the primary studies and the review finding, and the finding reflects the complexity and variation of the data, however only one study contributed to this finding) | Serious concerns (The finding is supported by data from one study and there are sufficient data to support it.) | | No or minor concerns the finding was relevant to the review question (population, context, setting) | Very Low | This finding was found in one study with some minor methodological limitations and some moderate concerns about coherence and serious concerns regarding adequacy of data and no concerns regarding relevance Therefore there is very low confidence in the use of this finding across all studies | |
| Social isolation | Elissa *et al.* 2017, Khandan *et al.* 2018 (b), reported this finding, n=25 participants | Content analysis | Minor concerns about methodological limitations (related to the reflexivity of the researcher as to their influence on the participants and vice-versa was not clear and methods of recruitment | Minor concerns (There was a clear fit between the data from the primary studies and the review finding, and the finding reflects the complexity and variation of the data) | Serious concerns (The finding is supported by data from two studies and there are sufficient data to support it.) | | No or minor concerns the finding was relevant to the review question (population, context, setting) | Low | This finding was found in two studies with some minor methodological limitations and some minor concerns about coherence and serious concerns regarding adequacy of data and no concerns regarding relevance Therefore there is low confidence in the use of this finding across all studies | |
| Impact on social life | Elissa *et al.* 2017, Moghadam *et al.* 2022, reported this finding, n=30 participants | Content analysis | Minor concerns about methodological limitations (related to the reflexivity of the researcher as to their influence on the participants and vice-versa was not clear and methods of recruitment | Minor concerns (There was a clear fit between the data from the primary studies and the review finding, and the finding reflects the complexity and variation of the data) | Serious concerns (The finding is supported by data from two studies and there are sufficient data to support it.) | | No or minor concerns the finding was relevant to the review question (population, context, setting) | Low | This finding was found in two studies with some minor methodological limitations and some minor concerns about coherence and serious concerns regarding adequacy of data and no concerns regarding relevance Therefore there is low confidence in the use of this finding across all studies | |
| Social burden | | | | | | | | | | |
| Social stigma | Elissa *et al.* 2017, Rossiter *et al.* 2019, Asaad *et al.* 2022, reported this finding, n=25 participants | Phenomenology, content analysis, Yin's qualitative data analysis | Minor concerns about methodological limitations (related to the reflexivity of the researcher as to their influence on the participants and vice-versa was not clear and methods of recruitment | No or minor concerns (There was a clear fit between the data from the primary study and the review finding, and the finding reflects the complexity and variation of the data.) | Minor concerns (The finding is supported by data from three studies, with rich data to support it) | | No or minor concerns the finding was relevant to the review question (population, context, setting) | Moderate | This finding was found in three studies with some minor methodological limitations and minor concerns about coherence and adequacy of data and no concerns regarding relevance Therefore there is moderate confidence in the use of this finding across all studies | |
| Social discrimination | Elissa *et al.* 2017, Rossiter *et al.* 2019, n=14 participants | Content analysis, Yin's qualitative data analysis | Minor concerns about methodological limitations (related to the reflexivity of the researcher as to their influence on the participants and vice-versa was not clear and methods of recruitment | Minor concerns (There was a clear fit between the data from the primary studies and the review finding, and the finding reflects the complexity and variation of the data) | Serious concerns (The finding is supported by data from two studies and there are sufficient data to support it.) | | No or minor concerns the finding was relevant to the review question (population, context, setting) | Low | This finding was found in two studies with some minor methodological limitations and some minor concerns about coherence and serious concerns regarding adequacy of data and no concerns regarding relevance Therefore there is low confidence in the use of this finding across all studies | |
| Separation anxiety | Elissa *et al.* 2017, reported this finding, n=10 participants | Content analysis | Minor concerns about methodological limitations (related to the reflexivity of the researcher as to their influence on the participants and vice-versa was not clear and methods of recruitment | Moderate concerns (There was a clear fit between the data from the primary studies and the review finding, and the finding reflects the complexity and variation of the data, however only one study contributed to this finding) | Serious concerns (The finding is supported by data from one study and there are sufficient data to support it.) | | No or minor concerns the finding was relevant to the review question (population, context, setting) | Very Low | This finding was found in one study with some minor methodological limitations and some moderate concerns about coherence and serious concerns regarding adequacy of data and no concerns regarding relevance Therefore there is very low confidence in the use of this finding across all studies | |
| **Spiritual** | | | | | | | | | | |
| Spiritual role in accepting diagnosis | Six studies (Povlsen & Ringsburg 2008, Khandan *et al.* 2018 (a, b), Rossiter *et al.* 2019, Asaad *et al.* 2022, Momani *et al.* 2022) reported this finding. n=67 participants. | Grounded theory, Phenomenology, content analysis, Yin's qualitative data analysis | Minor concerns about methodological limitations (related to the reflexivity of the researcher as to their influence on the participants and vice-versa was not clear and methods of recruitment | No or minor concerns (There was a clear fit between the data from the primary study and the review finding, and the finding reflects the complexity and variation of the data.) | Minor concerns (The finding is supported by data from six studies, with rich data to support it) | | No or minor concerns the finding was relevant to the review question (population, context, setting) | Moderate | This finding was found in six studies with some minor methodological limitations and minor concerns about coherence and adequacy of data and no concerns regarding relevance Therefore there is moderate confidence in the use of this finding across all studies | |
| Spiritual role in coping | Povlsen & Ringsberg 2008, Rossiter *et al.* 2019, Asaad *et al.* 2022, Moghadam *et al.* 2022, reported this finding, n=42 participants. | Phenomenology, content analysis, Yin's qualitative data analysis | Minor concerns about methodological limitations (related to the reflexivity of the researcher as to their influence on the participants and vice-versa was not clear and methods of recruitment | No or minor concerns (There was a clear fit between the data from the primary study and the review finding, and the finding reflects the complexity and variation of the data.) | Minor concerns (The finding is supported by data from four studies, with rich data to support it) | No or minor concerns the finding was relevant to the review question (population, context, setting) | | Moderate | | This finding was found in four studies with some minor methodological limitations and minor concerns about coherence and adequacy of data and no concerns regarding relevance Therefore there is moderate confidence in the use of this finding across all studies |
| God’s will/destiny | Povlsen & Ringsberg 2008, Khandan *et al.* 2018 (b), Rossiter *et al.* 2019, Momani *et al.* 2022, reported this finding, n=45 participants | Grounded theory, content analysis, Yin's qualitative data analysis | Minor concerns about methodological limitations (related to the reflexivity of the researcher as to their influence on the participants and vice-versa was not clear and methods of recruitment | No or minor concerns (There was a clear fit between the data from the primary study and the review finding, and the finding reflects the complexity and variation of the data.) | Minor concerns (The finding is supported by data from four studies, with rich data to support it) | No or minor concerns the finding was relevant to the review question (population, context, setting) | | Moderate | | This finding was found in four studies with some minor methodological limitations and minor concerns about coherence and adequacy of data and no concerns regarding relevance Therefore there is moderate confidence in the use of this finding across all studies |
| Spiritual support | Moghadam *et al.* 2022, reported this finding, n=20 participants | Content analysis | Minor concerns about methodological limitations (related to the reflexivity of the researcher as to their influence on the participants and vice-versa was not clear and methods of recruitment | Moderate concerns (There was a clear fit between the data from the primary studies and the review finding, and the finding reflects the complexity and variation of the data, however only one study contributed to this finding) | Serious concerns (The finding is supported by data from one study and there are sufficient data to support it.) | No or minor concerns the finding was relevant to the review question (population, context, setting) | | Very Low | | This finding was found in one study with some minor methodological limitations and some moderate concerns about coherence and serious concerns regarding adequacy of data and no concerns regarding relevance Therefore there is very low confidence in the use of this finding across all studies |
